# Unraveling the ecological landscape of mast cells in esophageal cancer through single-cell RNA sequencing

**DOI:** 10.3389/fimmu.2024.1470449

**Published:** 2024-10-04

**Authors:** Shengyi Zhang, Xinyi Zhang, Zhikai Xiahou, Shunqing Zuo, Jialong Xue, Yi Zhang

**Affiliations:** ^1^ Department of Thoracic Surgery, Songjiang Hospital Affiliated to Shanghai Jiao Tong University School of Medicine, Shanghai, China; ^2^ Clinical Medical College, Southwest Medical University, Luzhou, China; ^3^ China Institute of Sport and Health Science, Beijing Sport University, Beijing, China

**Keywords:** single-cell RNA sequencing, mast cells, EGFR signaling pathway, prognostic model, esophageal cancer

## Abstract

**Background:**

Esophageal cancer (EC) is a major health issue, ranking seventh in incidence and sixth in mortality worldwide. Despite advancements in multidisciplinary treatment approaches, the 5-year survival rate for EC remains low at 21%. Challenges in EC treatment arise from late-stage diagnosis, high malignancy, and poor prognosis. Understanding the tumor microenvironment is critical, as it includes various cellular and extracellular components that influence tumor behavior and treatment response. Mast cells (MCs), as tissue-resident immune cells, play dual roles in tumor dynamics. High-throughput single-cell RNA sequencing offers a powerful tool for analyzing tumor heterogeneity and immune interactions, although its application in EC is limited.

**Methods:**

In this study, we investigated the immune microenvironment of EC using single-cell RNA sequencing and established a comprehensive immune profile. We also performed analysis of upstream transcription factors and downstream pathway enrichment to further comprehensively decipher MCs in EC. Besides, we performed knockdown experiments to explore the role of epidermal growth factor receptor (*EGFR*) signaling pathway in MCs-tumor cell interactions, highlighting its potential as a prognostic marker. Finally, we constructed a prognostic model for EC, which provided valuable suggestions for the diagnosis and prognosis of EC.

**Results:**

Our analysis identified 11 major cell types, of which MCs were particularly present in pericarcinoma tissues. Further grouping of the 5,001 MCs identified 8 distinct subtypes, including *SRSF7*-highly expressed MCs, which showed strong tumor preference and potential tumor-promoting properties. Moreover, we identified the key signaling receptor *EGFR* and validated it by in vitro knockdown experiments, demonstrating its cancer-promoting effects. In addition, we established an independent prognostic indicator, *SRSF7*+ MCs risk score (SMRS), which showed a correlation between high SMRS group and poor prognosis.

**Conclusion:**

These findings illuminate the complex interactions within the tumor microenvironment of EC and suggest that targeting specific MCs subtypes, particularly via the *EGFR* signaling pathway, may present novel therapeutic strategies. This study establishes a comprehensive immune map of EC, offering insights for improved treatment approaches.

## Graphical Abstract

Article research flow chart. First, we downloaded the clinical data of three esophageal cancer patients from TCGA, and after a series of data processing, we obtained the UMAP map of esophageal cancer. After that, we focused on MCs to get its UMAP map and performed trajectory analysis, enrichment analysis, cellular communication analysis and transcription factor analysis sequentially. Finally, we performed *in vitro* experiments on the pathway derived from communication analysis, verified the effect of the pathway on esophageal cancer progression through a series of experiments, and analyzed the related prognosis, and the results obtained were consistent with our study.

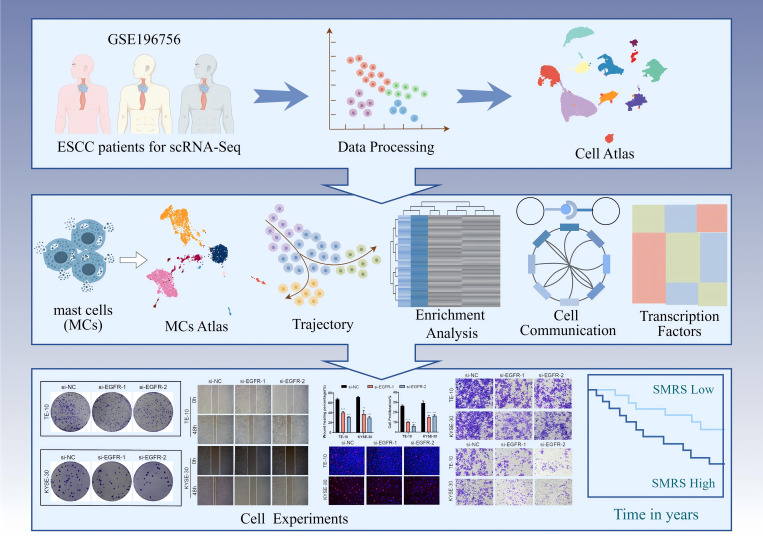

## 1 Introduction

Esophageal cancer (EC) is a common malignant tumor of the gastrointestinal system, with the seventh highest incidence and sixth highest mortality rate in the world (1). In China, the incidence and mortality rates of EC rank third and fourth, respectively, among all malignant tumors (2). Despite the development of a multidisciplinary treatment approach, the prognosis remains unfavorable (3). The 5-year survival rate for EC is only 21%, after pancreatic and liver cancers (4). Therefore, EC has been a major malignant tumor threatening the health of Chinese residents. EC consists of two main subtypes, esophageal squamous cell carcinoma (ESCC) and esophageal adenocarcinoma, with ESCC accounting for about 90% of all EC cases worldwide (5). EC is an aggressive cancer with rapid growth and a high rate of lymph node metastasis, usually involving the upper two thirds of the esophagus (6). In retrospective studies in EC, smoking, hot tea consumption, red meat consumption, poor oral health, low intake of fresh fruits and vegetables, and low socioeconomic status were associated with a higher risk of EC (7). Previous studies have shown that chronic inflammation plays a central role in progression from esophageal precancerous lesions (EPL) to esophageal squamous cell carcinoma, that dietary inflammatory potential has been linked to both EPL and ESCC, and that inflammatory imbalances promote tumorigenesis, and that the consumption of anti-inflammatory foods may be helpful in the prevention of EPL and ESCC (8–10). Difficulty swallowing and swollen lymph nodes in the neck do not appear until the cancer has progressed to an advanced stage (11), and the treatment of EC patients faces major challenges due to the lack of early symptoms, high malignancy, poor prognosis, and surgical complexity of EC. Although we have made great progress in the treatment of EC in recent years, especially through preoperative radiotherapy combined with immunotherapy, which shows a broad potential in the treatment of EC. However, due to the high rate of post-treatment recurrence and the limitations of drugs and treatment strategies after metastasis, only a small proportion of EC patients can benefit from the available treatments, while the majority of patients respond poorly to the treatments, and therefore, the overall survival rate of EC is still disappointing in China (3, 12).

In addition, due to the heterogeneity and complexity of tumors, the mechanisms of tumor proliferation, metastasis, drug resistance, and immunosuppression are unknown. Therefore, elucidating the molecular mechanisms of tumorigenesis and tumor progression is crucial for effective control and management of tumor development. Notably, the presence of non-tumor cells within the tumor tissue is also critical for tumor development (13). Therefore, shifting the therapeutic focus to other components of the tumor microenvironment (TME) may become an important strategy for future tumor therapy. The introduction of TME has played a very powerful role in advancing oncology research. TME has had an incredibly important role in the development and evolution of EC (14). The TME consists of multiple cellular components (e.g., fibroblasts, endothelial cells, and immune cells) and extracellular components (including cytokines, hormones, extracellular matrices, and growth factors), which form a complex network that encapsulates EC cells. These cells shape cancer biology and influence the response to treatment (15–17). In TME, mast cells (MCs) are tissue-resident immune cells that are important players in diseases associated with chronic inflammation such as cancer. Because MCs can infiltrate solid tumors and promote or limit tumor growth, MCs may polarize to either pro- or anti-tumor phenotypes and remain a challenging area of research (18). Previous articles have also hypothesized that *NRF2* in combination with AC-MCs may be a predictive marker for prognosis and may influence immunotherapy by modulating PD-L1 in EC (19).

High-throughput single-cell RNA sequencing (scRNA-seq), developed in recent years, is an effective method that has been shown to dissect heterogeneous tumors and decipher the interactions between cancer cells and their microenvironmental components, and to elucidate the transcriptomic profiles of both the cancer cells and the microenvironmental components (20–22), which is the basis and foundation for furthering the understanding of cancers and the development of effective early diagnostic and therapeutic strategies, previous studies have dissected the esophageal squamous cell carcinoma ecosystem by single-cell transcriptomic analysis (16), but its application in EC is still limited. At the same time, there is still a long way to go for early detection of esophageal cancer (23), and prognostic tools lack the necessary accuracy to facilitate individualized patient management strategies (24).

Therefore, in this study, scRNA-seq was used to sequence EC samples in order to decipher the immune microenvironment of EC, reveal the immune map of EC, and provide new insights for the treatment of EC. The functional role of MCs subtypes in EC and their association with tumor tissues are extensively discussed and summarized in this paper, and a prognostic model is established, which provides a valuable resource for deeper understanding of the causes and progression of EC and helps to improve its therapeutic strategies.

## 2 Materials and methods

### 2.1 Data source

The scRNA-seq data of EC were acquired from the GEO website (https://www.ncbi.nlm.nih.gov/geo/) under the accession number GSE196756. Patient clinical sample information can be found at https://www.ncbi.nlm.nih.gov/geo/query/acc.cgi. Considering the utilization of publicly accessible data derived from databases, it was not required to secure an ethical endorsement for this investigation.

### 2.2 Single‐cell sequencing

The gene expression data were imported into the R software and analyzed using the Seurat R package (25, 26). Cells of inferior quality were excluded based on the following criteria (1): nFeature between 300 and 7,500 (2); nCount between 500 and 100,000 (3); mitochondrial gene expression occupying no more than 25% of the total gene count within the cell (4); erythrocyte gene expression not surpassing 5% of the total gene count within the cell.

Subsequently, all gene expression data underwent normalization and scaling using the “NormalizeData” and “ScalData” functions within the Seurat R package (27). For the purpose of principal component analysis, the “FindVariableFeautres” function (28) was implemented to identify the top 2,000 most variable genes. These cells were then segregated into clusters based on the top 30 principal components (PCs) using the “FindClusters” function at a resolution of 1.0. Finally, the top 30 significant PCs were selected to dimensionality reduction and visualization of gene expression through uniform manifold approximation and projection (UMAP) (29, 30). The harmony R package (31, 32) was employed to alleviate the influence of batch effects among the samples. The dim value was set to 30, while the resolution parameter was configured to 1.2.

### 2.3 Identification of cell subtypes

Cell clusters were initially discerned utilizing the “FindClusters” and “FindNeighbors” functions within Seurat (33–35), employing a default resolution of 0.8. Afterwards, these cell clusters were bestowed with annotations based on the average gene expression of representative markers. In order to evaluate differentially expressed genes (DEGs) across distinct cell clusters, a Wilcoxon rank sum test was employed utilizing Seurat’s “FindAllMarkers” function (36, 37). The parameters min.pct and min.diff.pct were established at 0.25, while the LogFc threshold was set to 0.25.

### 2.4 Cancer preferences analysis

In order to evaluate the predilection of MCs subtypes for cancer, odds ratios were computed utilizing the calculation methodology (38).

### 2.5 Trajectory analysis of MCs subtypes

The slingshot R package was employed to deduce cellular lineages and pseudotimes. It delineated the structure of lineages through clustering-based minimum spanning trees and employed synchronized master curves to model branching trajectories for these lineages. The “getCurves” function was utilized to acquire refined trajectory curves. The association between gene expression and pseudotime was characterized by modeling the noise distribution of each gene through a generalized additive model with negative binomials. This approach allowed for the simulation of genes exhibiting a gradual alteration in expression throughout the pseudotime continuum (39).

### 2.6 Assessment of cell stemness

AUCell (40) represents a novel approach to discerning cells harboring active genes within single-cell RNA-seq datasets. Given a gene set as input, it provides an evaluation of the “activity” exhibited by that particular gene set in each individual cell. In the context of this study, AUCell was employed to quantitatively assess the level of stemness exhibited by various subtypes of MCs. To hypothesize the temporal trajectory of cell differentiation, the CytoTRACE R package was utilized (41).

### 2.7 Enrichment analysis of cellular subtypes

By leveraging the Gene Ontology (GO), Kyoto Encyclopedia of Genes and Genomes (KEGG), and Genome Enrichment Analysis (GSEA) tools, available at http://software.broadinstitute.org/gsea/msigdb, within the Cluster Profiler R package (42–44), we carried out enrichment analysis on the DEGs. To discern the disparities among various risk groups within the bulk data, the DESeq2 R package was applied, employing a threshold of |logFC| > 2 and a p-value threshold below 0.05.

### 2.8 Cell communication analysis

The CellChat R package (45) was used to analyze complex cell-to-cell interactions and develop regulatory networks based on ligand-receptor expression. The “netVisual DiffInteraction” function was applied to depict differences in communication strength among cells, and the “IdentifyCommunicationPatterns” function was utilized to estimate the number of communication patterns. A significance threshold of 0.05 was set. Various visualizations, including circle plots, bubble plots, and violin plots, were used to represent the incoming and outgoing signals of all cells

### 2.9 Scenic analysis

In evaluating the transcriptional activity within diverse subtypes of tumor cells, we employed the SCENIC analysis with Python.

### 2.10 Cell culture

Cell lines TE-10 and KYSE-30 were acquired from the American Type Culture Collection. The TE-10 cell line was grown under standard conditions (37°C, 5% CO_2_, 95% humidity) in RPMI1640 media with 10% fetal bovine serum (FBS) and 1% penicillin-streptomycin. KYSE-30 cell line was grown under standard conditions (37°C, 5% CO_2_, 95% humidity) in RPMI1640 media with 10% FBS, 1% penicillin-streptomycin, and 1% sodium pyruvate.

### 2.11 Cell transfection

*EGFR* knockdown was accomplished through the use of GenePharma (Suzhou, China) small interfering RNA (siRNA) constructs. According to Lipofectamine 3000 RNAiMAX (Invitrogen, USA) manufacturer’s instructions, transfection was carried out. Two knockdown constructs (Si-*EGFR*-1 and Si-*EGFR*-2) and a negative control (si-NC) were transfected into cells that had been plated at 50% confluency in six-well plates. Every transfection was carried out using Lipofectamine 3000 RNAiMAX (Invitrogen, USA).

### 2.12 Cell viability assay

Using the CCK-8 assay, the cell viability of transfected AGS and SGC-7901 cells was evaluated. After being cultivated for 24 hours, cells were planted at a density of 5×10³ cells per well in 96-well plates. Following the addition of 10μL of CCK-8 reagent (A311-01, Vazyme) to each well, the plates were incubated for two hours at 37°C in the dark. On days 1, 2, 3, and 4 post-transfections, absorbance at 450 nm was measured using a microplate reader (A33978, Thermo). Plotting of the mean OD values was done.

### 2.13 5-Ethynyl-2’-deoxyuridine proliferation assay

In 6-well plates, 5×10³ cells were planted per well with transfected CNE2 and HNE2 cells, and they were grown for an entire night. A 2x EdU working solution was then created by combining serum-free medium with 10 mM EdU. Following two hours of incubation at 37°C, the cells were rinsed with PBS, fixed for thirty minutes with 4% paraformaldehyde, permeabilized for fifteen minutes with a solution of 2 mg/mL glycine and 0.5% Triton X-100, then stained for thirty minutes at room temperature using a solution of 1X Apollo and 1X Hoechst 33342. The measurement of cell proliferation was done by fluorescence microscopy.

### 2.14 Wound-healing assay

In 6-well plates, stabilized transfected cells were plated and allowed to grow to confluence. Each well was scratched with a sterile 200μL pipette tip, and then it was cleaned with PBS to get rid of any remaining cell debris before being incubated in a medium without serum. Using Image-J software, the breadth of the scratches was measured after they were photographed at 0 and 48 hours.

### 2.15 Transwell assay

Before the experiment, cells were fasted for 24 hours in a serum-free medium. The upper chamber of Costar plates was filled with cell suspension after being treated with Matrigel (BD Biosciences, USA), while the lower chamber was filled with media containing serum. In a cell culture incubator, the cells were incubated for 48 hours. To evaluate the cells’ ability to invade, they were fixed with 4% paraformaldehyde after incubation and stained with crystal violet.

### 2.16 Construction and validation of the prognostic model

We determined the most important predictive genes using LASSO regression analysis and univariate Cox analysis (46–48). The risk coefficients for each prognostic gene were then determined using multivariate Cox regression analysis, allowing for the creation of a risk score model:


$$\text{Risk score=}\sum_{\text{i}}^{\text{n}}\text{Xi}\times \text{Yi}$$

X stands for the coefficient and Y represents the gene expression level. Using the “surv-cutpoint” function to compute the best cutoff value, patients were divided into two groups: low-risk and high-risk. We also used the Survival R package for survival analysis of the created risk score model and the “ggsurvplot” function (27) to depict survival curves in order to observe the prognostic outcomes in various patient cohorts. ROC curves were plotted using the timeROC R package to assess the predictive model’s accuracy and calibration (49, 50).

### 2.17 Kaplan-Meier survival curve of selected genes

We performed a survival analysis utilizing the R packages survminer and survival. The area under the ROC curve (AUC) was calculated after generating ROC curves for 1-year, 3-year, and 5-year survival rates using the Survive and Time ROC R packages. Model validation was conducted through survival analysis and time-dependent ROC analysis. To evaluate the model, we employed a heatmap, a scatter plot of survival status, and a distribution of risk scores.

## 3 Result

### 3.1 ScRNA sequencing revealed the main cell types in the EC

**Figure 1:**
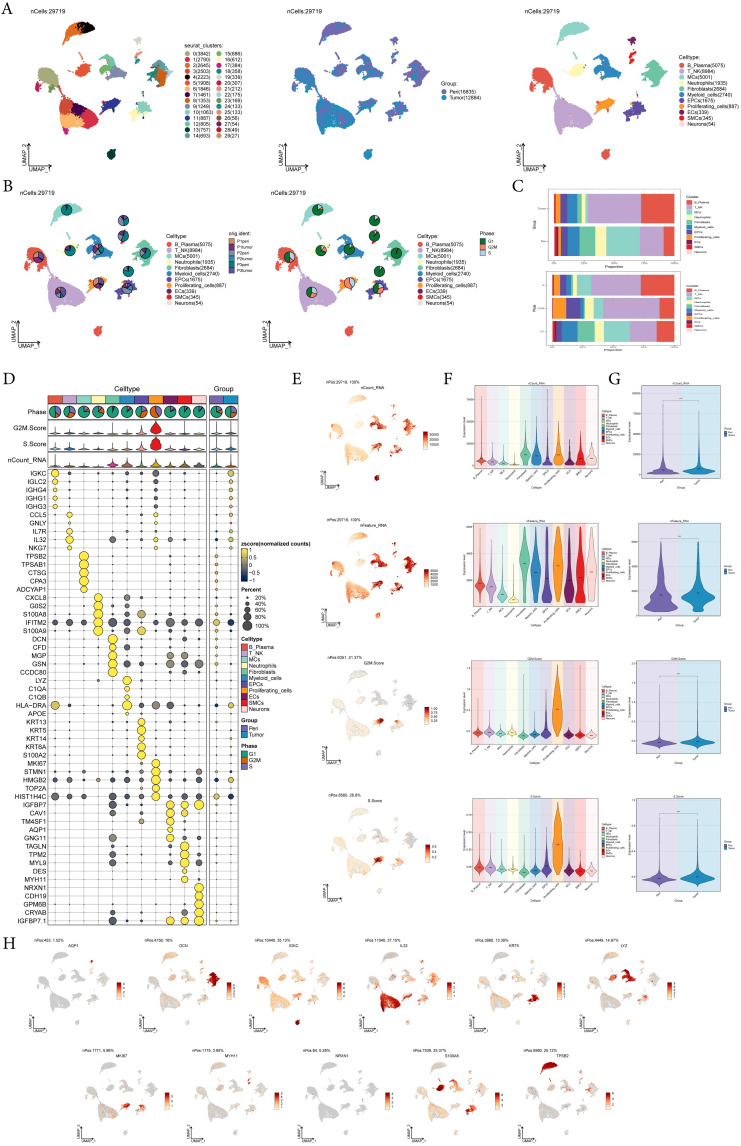
A single-cell profiling of EC, comprising 30 clusters and 11 cell types. **(A)** UMAP plot showed the 30 clusters of cells in EC patients and the number of cells in each cluster (left); UMAP plot showed the distribution of sample sources in the 11 cell types (middle); UMAP plot showed the 11 major cell types obtained by dimensionality reduction clustering of cells in EC (right). Each point corresponded to a single cell colored according to cell cluster or cell type. **(B)** The pie charts showed the proportion of different patient sources (left) and cell phases (right) in each cell type. **(C)** The bar graphs showed the proportion of different cell types in sample sources (top) and cell phase(bottom) respectively. **(D)** Bubble plot showed differential expression of top5 maker genes in EC cells across 11 cell types. Bubble colors were based on normalized data and sizes indicated the percentage of genes expressed in each cell type. **(E-G)** UMAP and violin plots revealed the expression levels of nCount-RNA, nFeature-RNA, G2M.Score, and S.Score in different cell types and sample sources. ****, p < 0.0001 indicated a significant difference. **(H)** UMAP plots visualized the differential genes of 11 cell types.

To identify the major cell types during the progression of EC, we collected pericarcinoma and tumor tissue samples from three EC patients for single-cell RNA sequencing (scRNA-seq). We also checked the quality and completeness of the raw data. This included checking for missing values, outliers, or any anomalies that might affect the analysis. We excluded genes in the sample that did not meet the minimum expression threshold. For example, genes with low counts or low variability were excluded as they may not provide meaningful insights. After performing initial quality control and removing batch effects, we retained a total of 29,719 cells. We categorized these 29,719 cells into 30 cell clusters by dimensionality reduction (**Figure 1A**). According to the cell gene map and typical markers, 30 cell clusters were finally identified into 11 cell types, including B-Plasma cells *(IGKC*), T-NK cells (*IL32*), mast cells (MCs, *TPSB2*), neutrophils (*S100A8*), fibroblasts (*DCN*), myeloid cells (*LYZ*), epithelial cells (EPCs, *KRT5*), proliferating-cells (*MKI67*), endothelial cell (ECs, *AQP1*), smooth muscle cell (SMCs, *MYH11*), and neurons (*NRXN1*). From the pie charts and bar graphs, we could learn that for tissues, T-NK cells accounted for the largest proportion in tumor tissues, followed by B-Plasma cells, while MCs were the most predominant cell type in pericarcinoma tissues; for phases, T-NK accounted for the largest proportion in both the G2M and the S phases, while on the contrary, most of MCs accounted for the largest proportion in the G1 phase (**Figures 1B, C**). **Figure 1D** showed the top 5 marker genes for 11 cell types. UMAP and violin plots were utilized to visualize nCount-RNA, nFeature-RNA, G2M.Score, and S.Score across all cells, demonstrating that proliferating cells exhibited the highest proliferative activity and vigorous division (**Figures 1E-G**). At the same time, the distribution of marker genes on UMAP for each cell type was presented (**Figure 1H**).

Among all cell types, MCs drew our attention. MCs play a crucial role in allergic reactions, pathogen immune responses during infections, angiogenesis, and the regulation of both innate and adaptive immunity. In addition to all these roles, MCs were increasingly recognized as regulators of the tumor microenvironment. Despite the accumulating evidence for MCs in tumors, their exact role in the tumor microenvironment remained incompletely understood (51). Therefore, we next performed a further analysis of mast cells.

### 3.2 Visualization of MCs subtypes in EC

**Figure 2:**
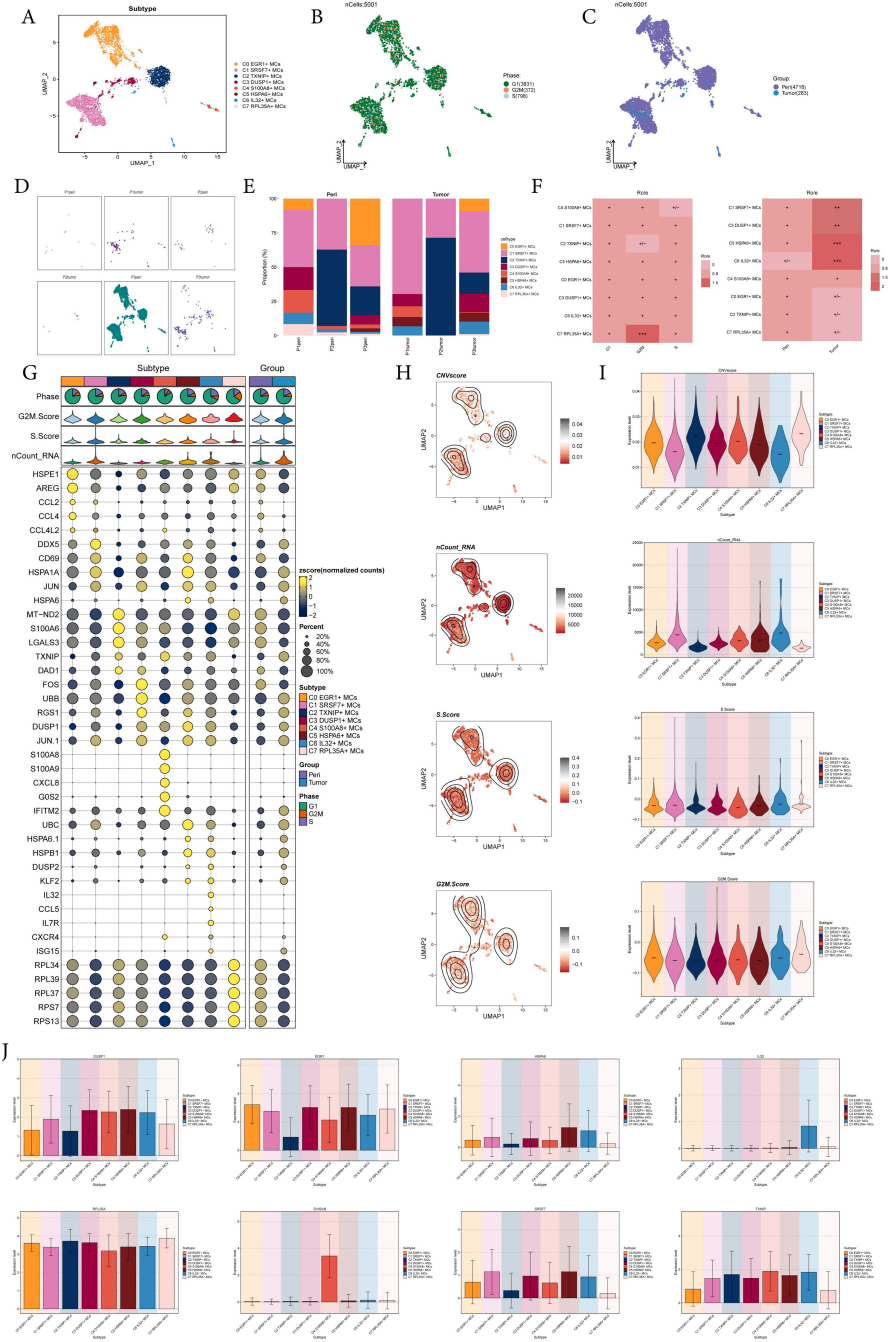
8 subtypes of MCs were identified with different markers. **(A-C)** UMAP plot demonstrated the 8 cell subtypes of MCs in EC patients and the number of cells in each cell subtype **(A)**; UMAP plot demonstrated the distribution of cell phases and sample sources in the 8 MCs subtypes respectively **(B, C)**. Each point corresponded to a single cell colored according to cell different groups. **(D)** UMAP plots showed the distribution of MCs in each patient source respectively. **(E)** The bar graphs showed the proportion of different MCs subtypes in each patient source. **(F)** Cell phases and sample sources preference of each MCs subtype estimated by Ro/e score. **(G)** Bubble plot showed differential expression of top 5 maker genes in 8 MCs subtypes. Bubble colors were based on normalized data and sizes indicated the percentage of genes expressed in each subtype. **(H, I)** UMAP and violin plots revealed the expression levels of CNVscore, nCount-RNA, S.Score and G2M.Score in different MCs subtypes. **(J)** Bar plots showed the expression levels of gene markers in each MCs subtype.

Next, we analyzed the scRNA-seq data from tumor and pericarcinoma tissues, identified MCs, and performed further sub-clustering. This analysis resulted in eight distinct cell subtypes from a total of 5,001 mast cells: C0 *EGR1*+ MCs, C1 *SRSF7*+ MCs, C2 *TXNIP*+ MCs, C3 *DUSP1*+ MCs, C4 *S100A8*+ MCs, C5 *HSPA6*+ MCs, C6 *IL32*+ MCs, C7 *RPL35A*+ MCs (**Figure 2A**), and showed the distribution of phases and sample sources in the subtypes, while faceting gived a clearer picture of the distribution of MCs from different sample sources (**Figures 2B-D**). The bar graphs illustrated that the C1 *SRSF7*+ MCs had the highest proportion of tumor tissues of P1 and P3 origin and was enhanced over the pericarcinoma tissues share, and similarly, the Ro/e preference graph corroborated this, suggesting that the C1 *SRSF7*+ MCs was more preferred to tumor tissues (**Figures 2E, F**). In order to better explore the characteristics of different MCs subtypes, we visualized their typical genes. As shown in **Figure 2G**, C1 S*RSF7*+ MC highly express *DDX5*, which had been shown to be associated with a variety of key tumor promoting molecular interactions and was involved in tumorigenesis and tumor progression signaling pathways (52). This suggested that C1 *SRSF7*+ MCs in EC might be involved in tumor promoting effect. Several related features (CNVscore, ncount-RNA, S.Score and G2M.Score) of eight MCs subtypes were visualized (**Figures 2H, I**). From the Figures, we could learn that C7 *RPL35A*+ MCs had the highest expression level of CNVscore and G2M.Score, while C1 *SRSF7*+ MCs and C6 *IL32*+ MCs had the highest nCount-RNA expression level, and all subtypes had basically the same expression level of S.Score. In the end, bar plots showed the expression level of gene makers in each MCs subtype, validating the basis for delineating subtypes (**Figure 2J**).

### 3.3 Slingshot analysis of proposed temporal trajectories of MCs subtypes

**Figure 3:**
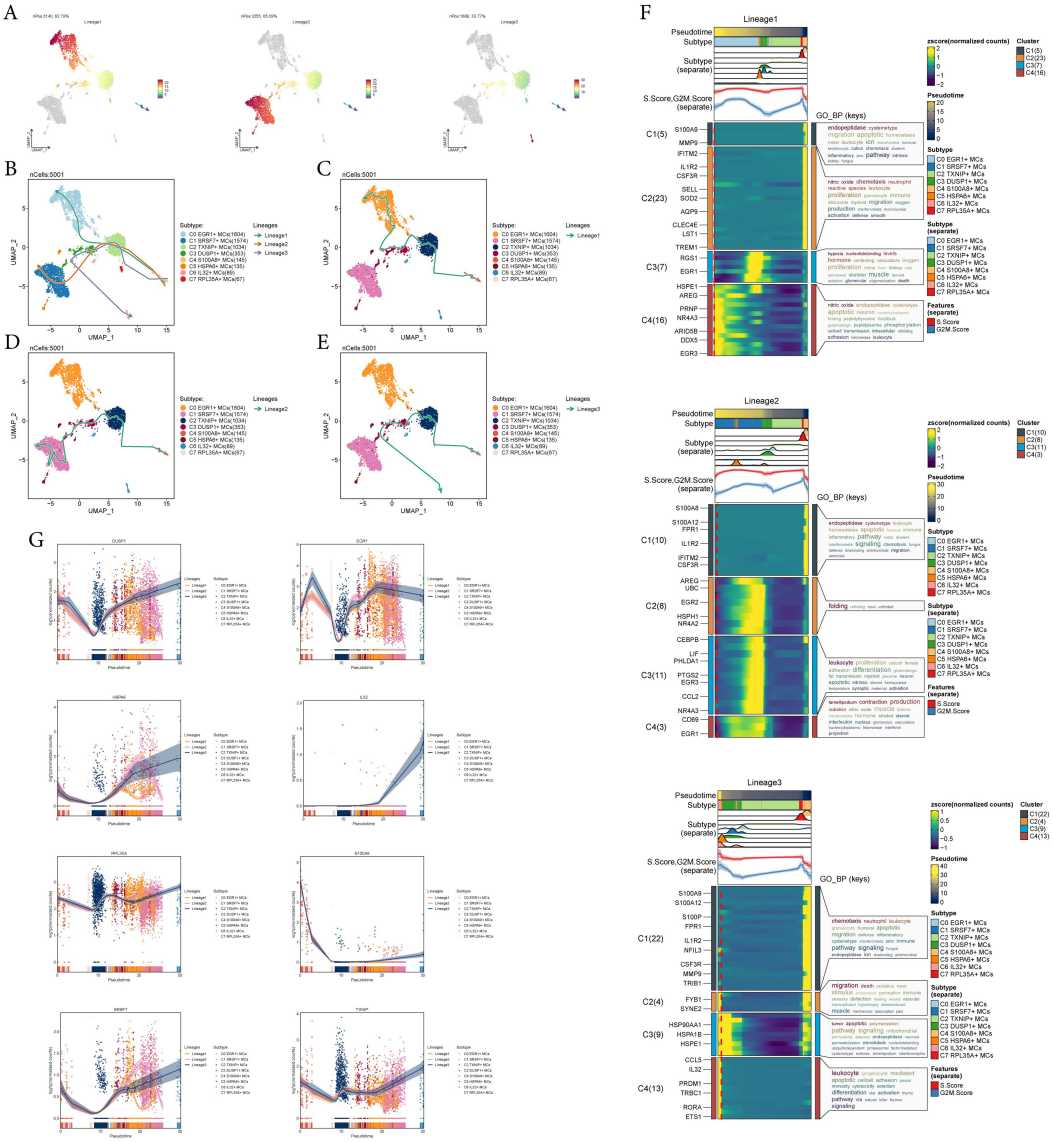
Slingshot analysis reveals three differentiation trajectories of MCs. **(A)** Demonstration of the distribution of slingshot-predicted MCs differentiation trajectories among all MCs by UMAP plot. Plotting each spectrum according to the pseudotime value to infer the result, the color from blue to red indicates the pseudotime from naïve to mature, and the grey part of the cells represent not belonging to the lineage. **(B-E)** The distribution of three differentiation trajectories of 8 MCs subtypes fitted by the pseudotime order in all mast cells **(B)**. Each trajectory was displayed respectively in UMAP **(C-E)**. **(F)** Heatmap demonstrated the related characteristics of 3 pseudotime trajectory lineages of MCs. The value of pseudotime correlated with differentiation, where 0 indicates the start point and 20 is the end point. **(G)** Scatter plots demonstrated the trajectories of named genes of 8 cell subtypes of mast cells changing on three lineages obtained after slingshot visualization.

To infer the lineage trajectory and pseudotime sequence of MCs, we employed slingshot analysis to assess the distribution of MCs differentiation trajectories across all MCs, visually represented through UMAP plots (**Figure 3A**). Then, we found 3 cell lineage trajectories of the MCs subtypes (**Figures 3B-E**). Including: lineage 1: C4 → C2 → C3 → C0; lineage 2: C4 → C2 → C3 → C1; lineage 3: C4 → C2 → C3 → C6. Slingshot analysis revealed that the differences among the three trajectories mainly reside in the late stages. Combined with **Figures 3C-E** to determine, lineage 1’s endpoint was located in C0, which showed no preference for tumor tissue, lineage 3’s endpoint was located in C6, which had a very small number of cells and a low percentage of tumor tissue, while lineage 2’s endpoint was located in C1, which not only showed a preference for tumor tissue, but also had a high percentage of tumor tissue. Therefore, we concluded that lineage 2 represented the differentiation line of MCs associated with the tumor. In addition, we also noted that MCs are influenced by some cytokines or tumor cell-secreted proteins during development in TME, resulting in a possible transformation of the MCs phenotype to a tumor-associated or pro-tumorigenic phenotype (18), whereas C1 belonged to the terminal end and consisted predominantly of MCs originating from tumor tissues, and based on this observation, we hypothesized that C1 may play a crucial role in the differentiation of tumor-associated MCs(TAMCs) process. Subsequently, we confirmed the biological processes corresponding to the three cell lineage trajectories of MCs subtypes using GO-BP enrichment analysis (**Figure 3F**). It was found that C1 in lineage 2 was associated with biological processes such as endopeptidase and cysteinetype, C2 was linked to processes such as protein folding, C3 was related to leukocyte functions, and C4 was involved in processes such as lamellipodium formation, contraction, and production. Finally, the dynamic trends plot demonstrated the expression variation and distribution of marker genes for MCs subtypes along the three differentiation trajectories in pseudotime (**Figure 3G**).

### 3.4 Expression of stemness gene sets in subtypes of MCs

**Figure 4:**
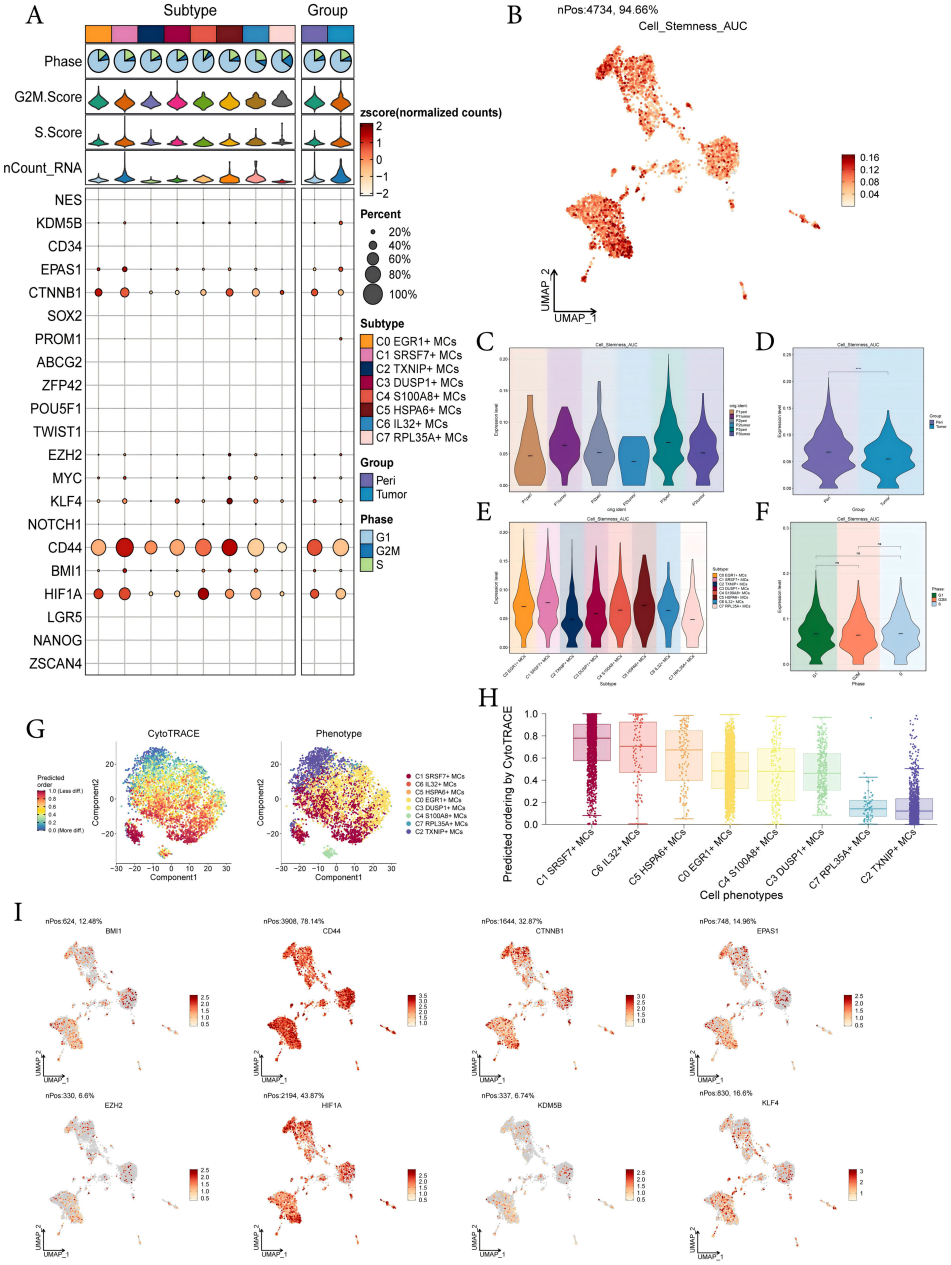
Analysis of cell stemness in mast cell subtypes. **(A)** Bubble plot showed expression of stemness genes in 8 MCs subtypes. Bubble colors were based on normalized data and sizes indicated the percentage of genes expressed in each subtype. **(B)** UMAP plot visualized the AUC values of cell stemness. **(C-F)** Violin plots revealed the expression levels of AUC values ​​of MCs cell stemness in different patient sources **(C)**, sample sources **(D)**, subtypes **(E)** and phases **(F)**. ****,p < 0.0001 indicated a significant difference, ns indicated a non-significant difference. **(G)** The left panel demonstrated the distribution of MCs CytoTRACE scores. The color represented high or low cell stemness. The right panel indicated the distribution of MCs subtypes. The color represented different MCs subtypes. **(H)** Box line plot ranked the stemness of MCs subtypes according to CytoTRACE. **(I)** UMAP plots visualized the 8 stemness genes expressed in MCs subtypes.

To investigate the expression of stem cell genes in MCs subtypes and to understand their differentiation potential, we used bubble plots to illustrate the different expression of stem cell genes in MCs subtypes. The results showed the corresponding expression of stem cell genes *KDM5B*, *EPAS1*, *CTNNB1*, *EZH2*, *KLF4*, *CD44*, *BMI1*, and *HIF1A* in MCs subtypes and different tissue types (**Figure 4A**). Subsequently, we visualized the cell stemness AUC scores of different MCs subtypes using a UMAP plot (**Figure 4B**). We then combined this with other analyses to assess the expression levels of stemness-related genes in different subtypes of MCs, and violin plots showed the different expression levels of stemness genes in different sample sources, tissues, subtypes of MCs, and phases, respectively (**Figures 4C-F**). The results showed that C1 *SRSF7*+ MCs exhibited a higher level of cell stemness, indicating a lower degree of differentiation and higher differentiated potential; and it also showed that pericarcinoma tissues had the higher level of cell stemness. In addition, there was no significant difference in the expression levels of stemness genes in different cell phases. By CytoTRACE analysis, C1 *SRSF7*+ MCs showed the lowest degree of differentiation and the highest cell stemness among all subtypes, which we hypothesized might be related to the transformation of MCs to TAMCs (**Figures 4G, H**). Afterwards, the expression profiles of stemness genes with relatively elevated expression levels in **Figure 4A** were demonstrated in all MCs by UMAP plots (**Figure 4I**).

### 3.5 Enrichment analysis of MCs subtypes in EC

**Figure 5:**
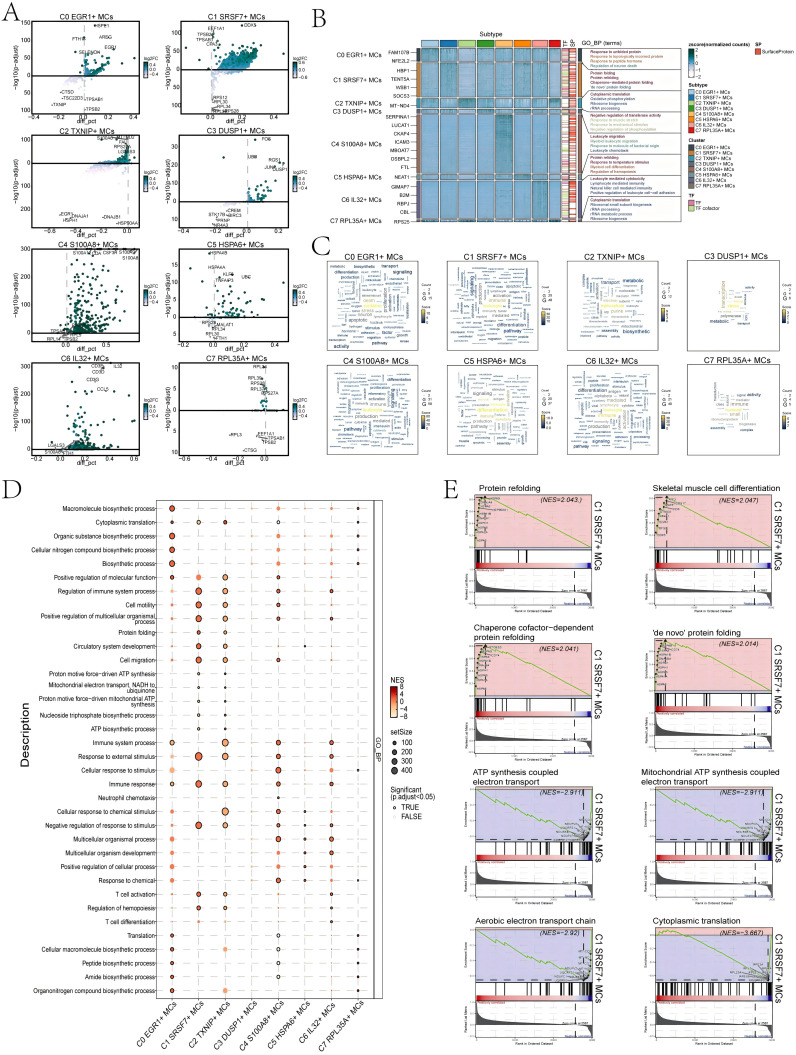
Results of functional enrichment analysis of differentially expressed genes in 8 MCs subtypes. **(A)** Volcano plots showed differentially expressed genes in 8 subtypes. **(B)** Heatmap showed the enrichment items of GO_BP scored. zscore > 0 was positive enrichment and < 0 was negative enrichment. **(C)** Word cloud diagrams demonstrated the activity of different pathways in MCs subtypes. **(D)** GSEA analysis diagram of different pathways in each MCs subtype. NES > 0 was positive enrichment and < 0 was negative enrichment. NES, N stands for standardization, and ES for enrichment scores. **(E)** GSEA enrichment analysis among C1 *SRSF7+* MCs.

First, we utilized volcano plots to represent the DEGs profiles between subtypes of MCs (**Figure 5A**). The results showed that the up-regulated DEGs in C1 *SRSF7*+ MCs were mainly *DDX5*, *EEF1A1*, *TPSB2*, *TPSAB1*, and *CPA3*. In addition, we performed GO-BP enrichment analysis of the DEGs in the subtypes of MCs to reveal their enrichment in biological processes. The heatmap showed the results of the top four enrichment entries in the MCs subtypes (**Figure 5B**). The C0 E*GR1*+ MCs subtype was mainly associated with pathways such as response to unfolded protein, response to topologically incorrect protein and regulation of neuron death; The C1 *SRSF7*+ MCs subtype was enriched in pathways such as protein folding, protein refolding, chaperone-mediated protein folding and ‘*de novo*’ protein folding; The C2 *TXNIP*+ MCs subtype revealed their close association with cytoplasmic translation, oxidative phosphorylation, ribosome biogenesis and rRNA processing; The C3 *DUSP1*+ MCs subtype showed enrichment in pathways such as negative regulation of transferase activity, response to muscle stretch, response to mechanical stimulus and negative regulation of phosphorylation; The C4 *S100A8*+ MCs subtype was enriched in pathways related to leukocyte migration, myeloid leukocyte migration, response to molecule of bacterial origin and leukocyte chemotaxis; The C5 *HSPA6*+ MCs subtype mainly exhibited enrichment in pathways such as protein refolding, response to temperature stimulus, myeloid cell differentiation and regulation of hemopoiesis; The C6 *IL32*+ MCs subtype revealed pathways related to leukocyte mediated cytotoxicity, lymphocyte mediated immunity, natural killer cell mediated immunity and positive regulation of leukocyte cell-cell adhesion; The enrichment analysis conducted on the C7 *RPL35A*+ MCs subtype revealed their association with cytoplasmic translation, ribosomal small subunit biogenesis, rRNA processing and rRNA metabolic process. The word cloud plots illustrated the enrichment results of DEGs across various pathways in the eight MC subtypes (**Figure 5C**). The results showed that the C1 *SRSF7*+ MCs subtype was mainly enriched in leukocyte, immune and activation, and it was hypothesized that C1 *SRSF7*+ MCs subtype might be related to MCs activation and participation in immune regulation.

In addition, the results of GSEA enrichment analysis were also shown in the form of bubble plots (**Figure 5D**). It showed that C1 *SRSF7+* MCs subtype was significantly expressed in regulation of immune system process, cell motility and migration, protein folding, response to immune and external stimulus pathways. All of the above pathways would suggest that MCs in the C1 *SRSF7*+ MCs subtype had likely transformed into TAMCs. Finally, we performed GSEA on the DEGs of the C1 *SRSF7*+ MCs subtype according to GO-BP terminology. The results were shown in **Figure 5E**. We observed that pathways associated with protein refolding, skeletal muscle cell differentiation, chaperone cofactor-dependent protein refolding and ‘*de novo*’ protein folding were upregulated in the C1 subtype. In contrast, pathways associated with ATP synthesis coupled electron transport, mitochondrial ATP synthesis coupled electron transport, aerobic electron transport chain and cytoplasmic translation were downregulated in the C1 subtype. Combining the above up-regulated genes and enriched pathways with previous studies, we believed that the C1 subtype was affected by the endoplasmic reticulum stress state, which disrupts the homeostasis of the original proteins and generates aberrant protein folding, and that this stress state could control a variety of pro-tumorigenic attributes of cancer cells, dynamically re-programming the function of immune cells, transforming MCs into TAMCs, thus exerting pro-tumorigenic effects, and conferring a greater tumorigenic, metastatic, and drug-resistant capacity to the malignant cells (53).

### 3.6 Transcription factors regulate the carcinogenic mechanism of C1 SRSF7+ MCs

**Figure 6:**
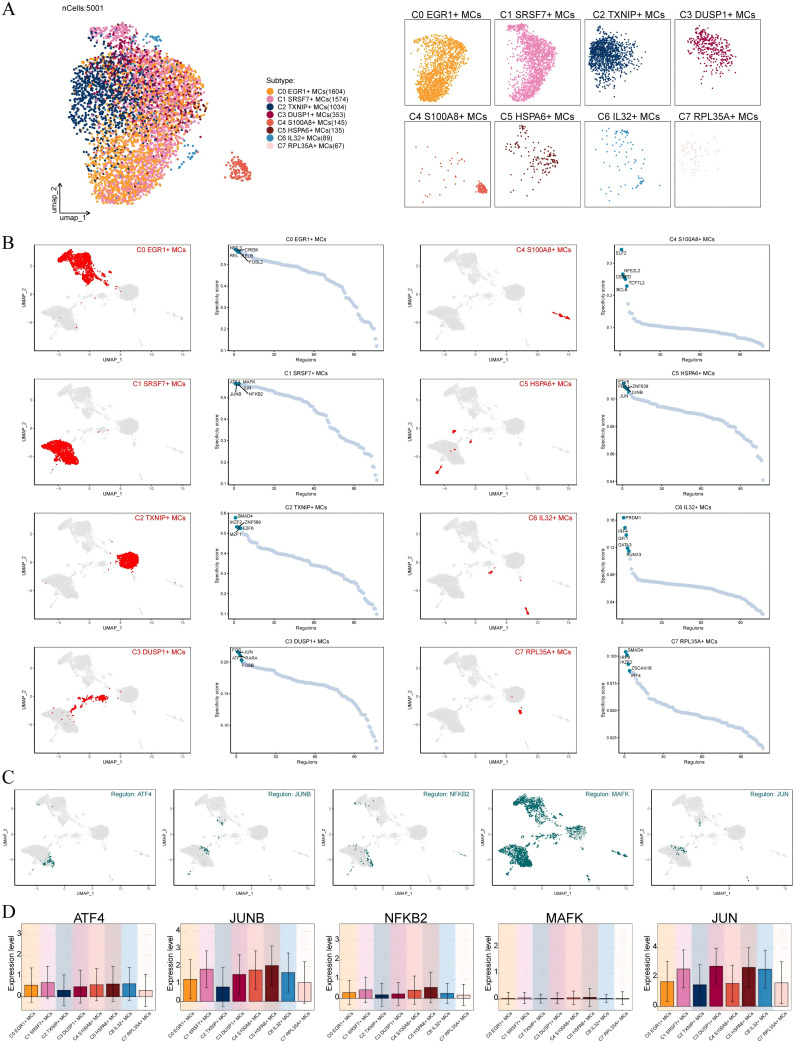
Identification of C1 SRSF7+ MCs Gene Regulatory Network. **(A)** UMAP visualized all MCs based on regulon activity score. Colored according to cell subtypes. **(B)** Different MCs subtypes were highlighted in the UMAP plots (red) (left); rank for regulators in different MCs subtypes based on regulon specificity score (RSS) (green) (right). **(C, D)** Expression of transcription factors ATF4, JUNB, NFKB2, MAFK and JUN of C1 *SRSF7+* MCs in different MCs subtypes.

Transcription factors can directly act on the genome and regulate gene transcription and affect the biological function of cells by combining specific nucleotide sequences in the upstream of the gene. Therefore, we used scenic to analyze the gene regulatory network of C1 *SRSF7*+ MCs. First of all, we carried out cluster analysis of MCs according to regulator activity (**Figure 6A**). It was obvious that the discretization of UMAP diagram based on regulator activity was smaller, the interference factors were better excluded, and all MCs were clustered and distributed. Among them, C1 *SRSF7*+ MCs were mainly distributed on the right side of UMAP plot without significant discretization. By further analyzing the key regulators of different MCs subtypes, the five major regulators of C1 *SRSF7*+ MCs, ATF4, JUNB, NFκB2, MAFK and JUN, were identified (**Figures 6B, C**). After analyzing these five key regulators in depth in conjunction with previous studies and **Figure 6D**, ATF4 and JUNB caught our attention. ATF4, which was expressed at higher levels in C1 *SRSF7*+ MCs than in other subtypes, was a major transcriptional regulator of the unfolded protein response to hypoxia, activated genes that promoted recovery of normal endoplasmic reticulum function and hypoxic survival (54), regulated mast cells through endoplasmic reticulum stress (55), and had been associated with programmed cell death in a variety of tumors, particularly ER stress-induced iron death (56, 57, 86). As for JUNB, its expression level was high in C1 *SRSF7*+ MCs, C4 *S100A8*+ MCs and C5 *HSPA6*+ MCs subtypes, and it is a potent inhibitor of endoplasmic reticulum stress and apoptosis, and, in particular, its modulation of endoplasmic reticulum stress is associated with ATF4 alterations (58).

### 3.7 CellChat analysis among all cells

**Figure 7:**
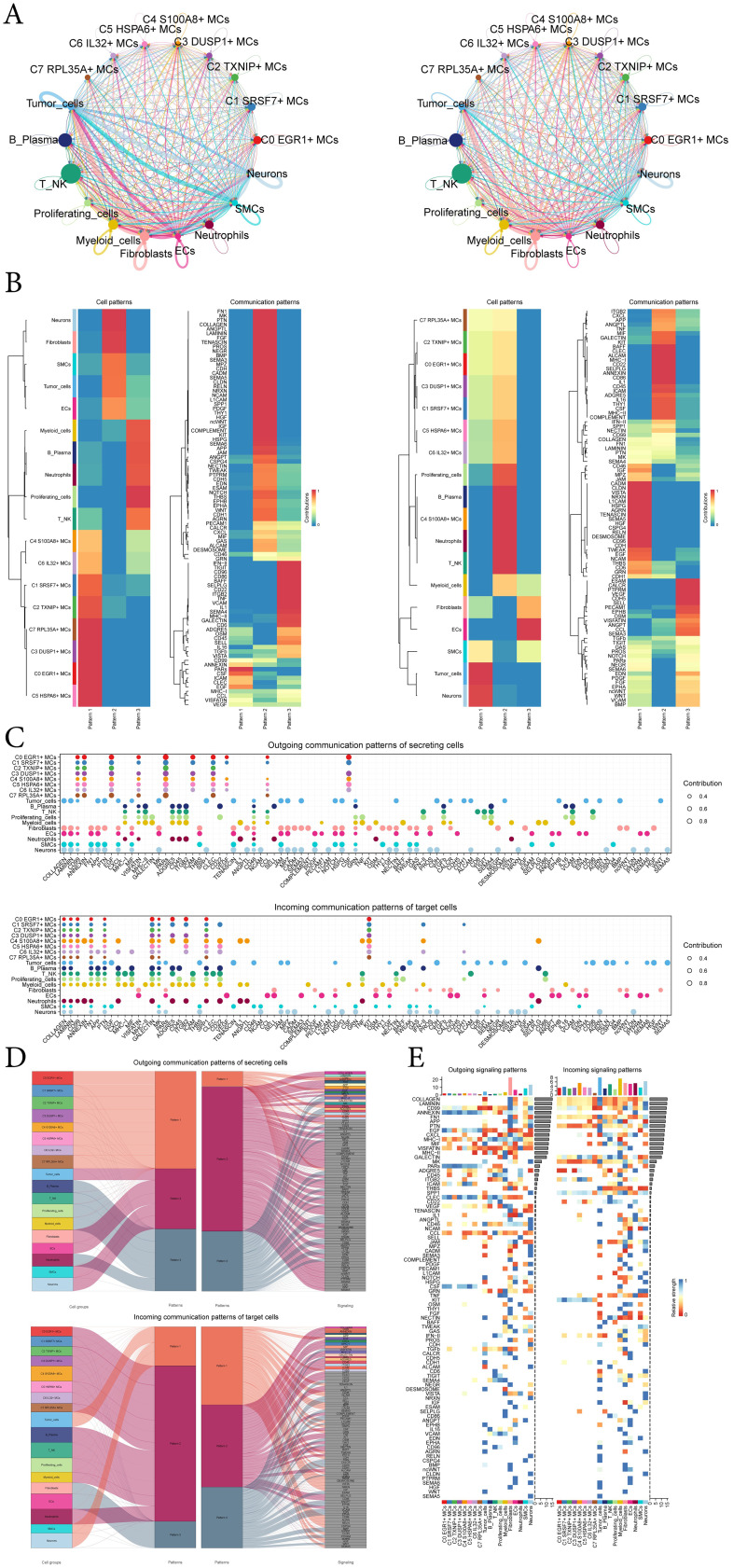
CellChat analysis among all cells. **(A)** Circle plots showed the number (left) and strength (right) of interactions between all cells. **(B)** Heatmap showed pattern recognition of outgoing cells (left), and incoming cells (right) among all cells. **(C)** Outgoing contribution bubble plot and incoming contribution bubble plot demonstrated the communication patterns between the secreting cells and target cells of EC, the color of the dots indicated different cells and the size of the dots indicated the contribution of cells. **(D)** Sankey diagrams showed inferred outgoing communication patterns of secreting cells and incoming communication patterns of target cells, as well as correspondence between inferred potential patterns, cell groups, and signaling pathways. The color and width of the branches represented the type and strength of the communication. **(E)** Heatmap showed ligands and receptors related to the incoming and outgoing signals of cell interactions.

In order to systematically elucidate complex cellular responses, we aimed to investigate cell-to-cell relationships and ligand-receptor communication networks to better understand interactions between cells. Using CellChat analysis, we initially established intercellular communication networks involving various cells such as tumor cells, fibroblasts, T-NK cells, and different subtypes of MCs, etc (**Figure 7A**). After establishing the intercellular communication networks using CellChat analysis, we calculated both the number of interactions (represented by the thickness of the connecting lines between two cell types) and the strength of interactions (indicated by the weight of the lines, where thicker lines denote stronger interaction strengths). This approach helped quantify the complexity and intensity of communication pathways between different cell types in the network. We utilized gene expression pattern analysis methods available through CellChat to investigate how cells and signaling pathways interact. Initially, we assessed the relationship between inferred potential communication patterns and groups of cells that secrete signaling molecules to decipher outgoing communication patterns. Three distinct signaling patterns were identified through our analysis: pattern 1 (subtypes of MCs), pattern 2 (Neurons cells, fibroblasts, SMCs, tumor-cells and ECs) and pattern 3 (myeloid-cells, B-Plasma cells, neutrophils, proliferating-cells and T-NK cells) (**Figure 7B**). To identify the key incoming and outgoing signals associated with the eight MCs subtypes, we quantitatively analyzed the ligand-receptor network using CellChat. This approach allowed us to predict the primary incoming signals from secreting cells (signal senders) releasing various cytokines or ligands. Additionally, we assessed which cell types acted as targeting cells (signal receivers), and how ligand-receptor-mediated communications between different cell types contributed to the progression of EC. This analysis helped illustrate how receptors on these cells were targeted by ligands released either from the same type of cell or from other cell types (**Figure 7C**).

In addition to examining detailed communication within individual pathways, an important aspect was understanding how multiple cell populations and signaling pathways coordinate their functions. To address this, CellChat employed a pattern recognition method based on nonnegative matrix decomposition. This method identified global communication patterns and key signals across different cell groups, shedding light on how various cells and pathways collaborate in their functions. The application of this analysis revealed three distinct incoming signaling patterns and three outgoing signaling patterns. For instance, this output indicated that the majority of outgoing MCs signaling was characterized by pattern 1, which represented multiple pathways, including but not limited to CD99, ANNFXIN, EGF, PARs, ICAM, CSF, etc. All output tumor-cells, fibroblasts, ECs, SMCs, neurons signalings were characterized by pattern 2, which represented pathways such as COLLAGEN, LAMININ, FN1, APP, PTN and so on. On the other hand, the analysis of communication patterns in target cells indicated that incoming signalings to tumor-cells, SMCs, and neurons were predominantly characterized by pattern 1. This pattern included signaling pathways such as EGF, TENASCIN, JAM, MPZ, CADM, and TWEAK. In contrast, the majority of incoming signalings to subtypes of MCs, B-plasma cells, T-NK cells, proliferating-cells, myeloid-cells, and neutrophils were characterized by pattern 2, which was driven by pathways such as CXCL and ANNEXIN (**Figure 7D**).

Combining the above analysis and the demonstration of all incoming and outgoing signal intensities in **Figure 7E**, the signaling molecule EGF caught our attention. EGF was present in the incoming pathway of tumor-cells, i.e., tumor-cells were the target cells, and EGF is again present in the outgoing pathway of C1 *SRSF7*+ MCs subtype, i.e. C1 *SRSF7*+ MCs subtype was the secreting cell, which links C1 *SRSF7*+ MCs subtype and tumor-cells, we speculated that this signaling pathway might be related to tumor progression, so we next focused on EGF.

### 3.8 Analysis of AREG-EGFR/AREG-(EGFR+ERBB2) signal pathway

**Figure 8:**
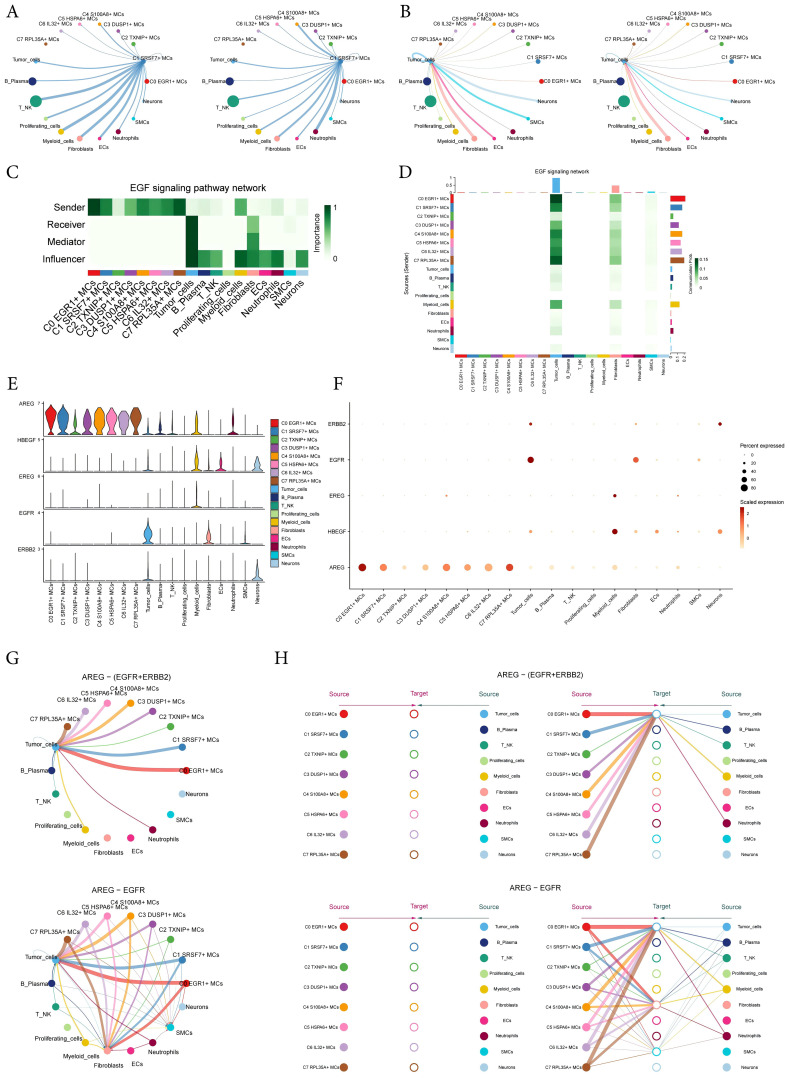
Visual analysis of AREG-EGFR/AREG-(EGFR+ERBB2) signaling pathway. **(A, B)** The number (left) and strength (right) of cellular interactions circled plots with C1 *SRSF7*+ MCs as source **(A)** and tumor as target **(B)**. **(C)** Heatmap demonstrated the centrality score of the EGF signaling pathway network, showing the relative importance of each cell group. **(D)** Heatmap showed the cell interactions of the EGF signaling pathway. **(E, F)** Violin and bubble plots demonstrated cellular interactions in the EGF signaling pathways. **(G–H)** Circle plot and hierarchical plots showed the inferred intercellular communication network for EGF signaling. Solid and hollow circles indicated source and target cell types in hierarchical plots, respectively. The edge color of the middle circle in hierarchical plots was consistent with the signal source.

The circular displayed the inferred cell-cell communication network between MCs and other cells (**Figures 8A, B**). The results showed that there was a strong crosstalk between C1 *SRSF7*+ MCs and tumor cells. We considered all identified cell types in ECEC as source cells for the EGF signaling pathway, and the results indicated that all subtypes of MCs could target tumor cells with released EGF. In addition to the senders and receivers of EGF signaling, based on the relative importance of each cell type in EGF signaling-mediated intercellular communication, we identified the cell types that act as mediators and influencers in this process, which is referred to as the “centrality measurement” algorithm. As can be seen from the Figure, C1 *SRSF7*+ MCs subtype had higher expression as a ‘sender’ in the EGF signaling pathway, whereas tumor-cells were acting as ‘receiver’, ‘mediator’ and ‘influencer’ in this signaling pathway (**Figure 8C**). Similarly, the heatmap corroborated this conclusion (**Figure 8D**). The violin plot showed the cell-cell interactions while giving the different ligands and receptors in the EGF signaling pathway, and the results showed that C1 *SRSF7*+ MCs subtype and tumor-cells were mainly contacted with *AREG* as a ligand and *EGFR* or *ERBB2* as receptors (**Figure 8E**). Bubble and circle plot as well as hierarchical plots likewise corroborated this conclusion (**Figures 8F–H**). Combined with the results of previous results in this paper, it can be concluded that the C1 *SRSF7*+ MCs and tumor cells crosstalk through the AREG-EGFR/AREG-(EGFR+ERBB2) signal pathway, thereby exerting a tumor-promoting effect.

### 3.9 *In vitro* experimental validation of EGFR

**Figure 9:**
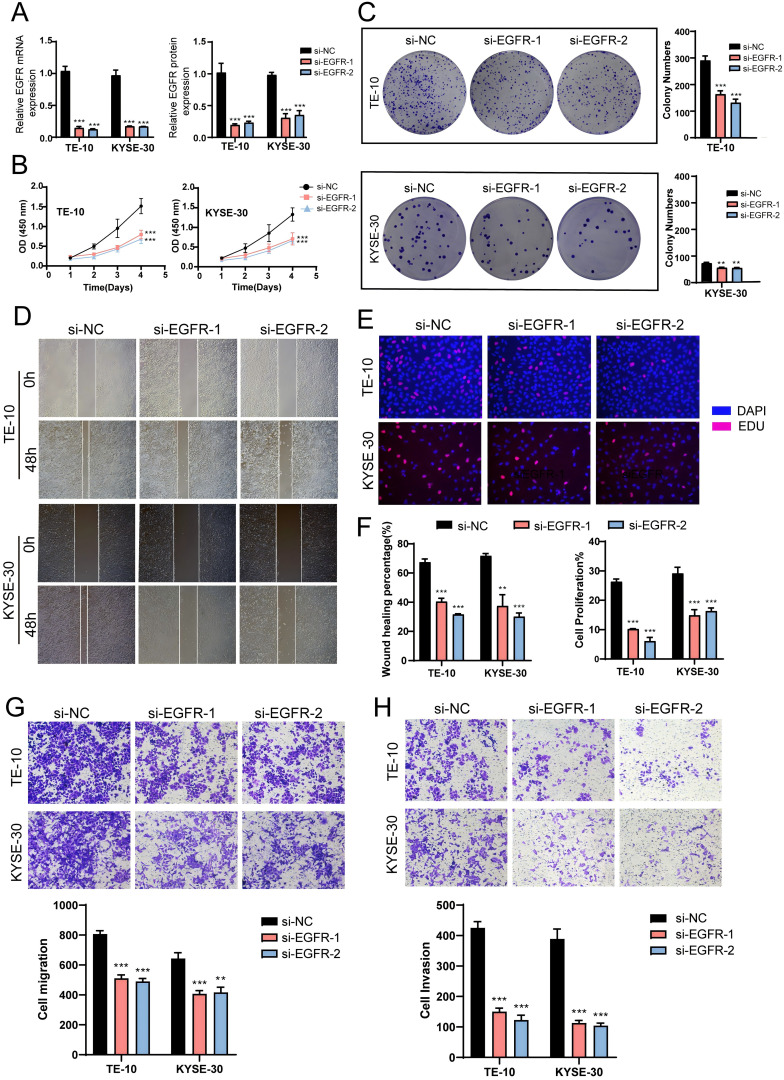
*In vitro* experiments confirmed the effects of *EGFR* knockdown. **(A)** Following *EGFR* knockdown, both mRNA and protein expression levels were significantly reduced. **(B)** The CCK-8 assay demonstrated a marked decrease in EC cell viability post-*EGFR* knockdown compared to the control group. **(C)** Colony formation assays revealed a significant reduction in colony numbers after *EGFR* knockdown. **(D)** The scratch assay indicated that *EGFR* knockdown inhibited EC cell migration. **(E)** The EDU staining assay confirmed that *EGFR* knockdown exerted an inhibitory effect on EC cell proliferation. **(F)** Bar graphs showed a significant reduction in both EC cell migration and proliferation capabilities post-*EGFR* knockdown (P < 0.01). **(G, H)** Transwell experiments indicated that *EGFR* knockdown inhibited the migration and invasion capabilities of tumor cells in the TE-10 and KYSE-30 cell lines. ***, p < 0.001; ****, p < 0.0001 indicates significant difference.

To further investigate the role of *EGFR* in EC, we conducted *in vitro* experiments using the TE-10 and KYSE-30 cell lines. Initially, we knocked down *EGFR* and measured the mRNA and protein expression levels before and after knockdown. We observed a significant reduction in both mRNA and protein expression levels in both cell lines compared to the control group (**Figure 9A**). Subsequently, the CCK-8 assay revealed a marked decrease in EC cell viability post-*EGFR* knockdown (**Figure 9B**). Colony formation assays and EDU experiments confirmed that *EGFR* knockdown inhibited EC cell proliferation (**Figures 9C, E, F**). Additionally, scratch and transwell assays were employed to assess the migration and invasion capabilities of EC cells post-*EGFR* knockdown, demonstrating a significant reduction in migration and invasion levels (**Figures 9D, F-H**). These results collectively indicate that *EGFR* knockdown suppresses the activity, proliferation, migration, and invasion of EC cells, thereby inhibiting tumor growth.

### 3.10 Enrichment analysis and construction of predictive models

**Figure 10:**
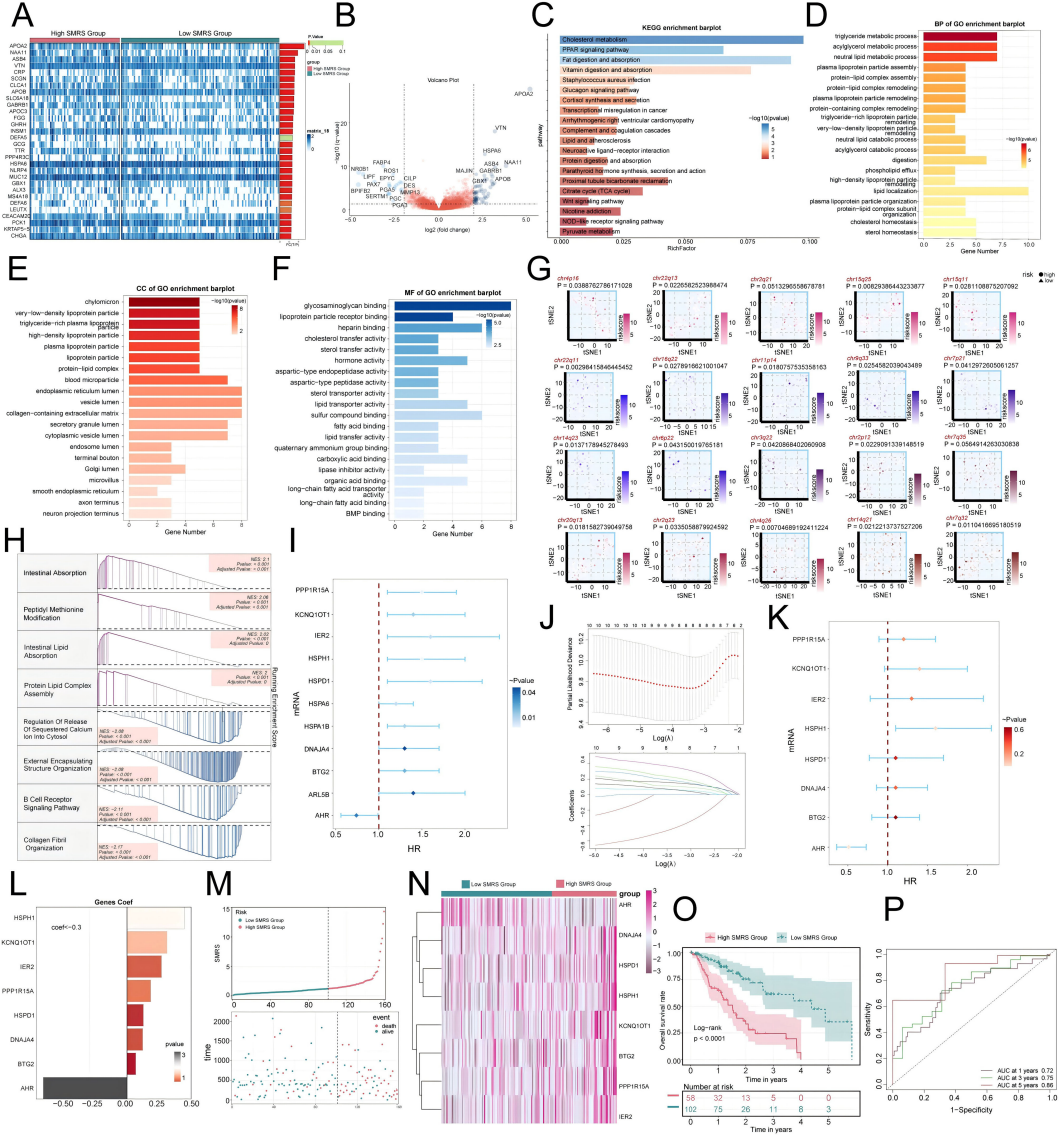
Enrichment analysis of differential genes and construction of the prognostic model. **(A)** Heatmap illustrated the expression profiles of differential genes in high and low SMRS groups. **(B)** Volcano plot depicted the distribution of differential genes in high and low SMRS groups. **(C-F)** Bar charts separately presented the enrichment analysis results of differential genes in KEGG, GO-BP, GO-CC, and GO-MF pathways for high and low SMRS groups. **(G)** t-SNE plot visualized the risk score distribution of the top-ranked GSVA enrichment term in high and low SMRS groups. **(H)** Detailed exposition of GSEA pathway enrichment results for differential genes across various pathways was provided. **(I)** Forest plot from univariate Cox regression analysis showcased statistically significant genes (P<0.05) with HR<1 indicating protective factors and HR>1 indicating risk factors. **(J)** Selection of eight prognostic-related genes (non-zero regression coefficients) was made via LASSO regression analysis, with optimal parameter (lambda) determined through cross-validation (top), and LASSO coefficient curve determined by optimal lambda (bottom). **(K)** Forest plot of eight prognosis-related genes. **(L)** Bar chart showed the Coef values of genes utilized for model construction. **(M)** Curve chart illustrated the risk scores of high and low SMRS groups, and scatter plot depicted survival/death events over time for both groups. **(N)** Heatmap displayed differential expression of model genes, with color scale based on normalized data. **(O)** Kaplan-Meier curves demonstrated survival disparities between high and low SMRS groups. **(P)** ROC curve and AUC value were used to evaluate the sensitivity and specificity of the prognostic model in predicting 1-year, 3-year and 5-year prognosis.

To further investigate the impact of MCs with high *SRSF7* expression on EC patients, we divided the TCGA cohort patients into high and low SMRS (SMRS: *SRSF7*+MCs risk score) groups according to the gene expression levels of the *SRSF7*+ MCs subtype. A heatmap illustrated the expression profiles of the top 30 DEGs (**Figure 10A**), and a volcano plot depicted the up-regulation and down-regulation of DEGs (**Figure 10B**). Subsequently, we employed various enrichment methods to gain insights into the associated biological processes. KEGG enrichment analysis revealed that DEGs were primarily enriched in pathways such as cholesterol metabolism, PPAR signaling pathway, and Fat digestion and absorption (**Figure 10C**). In GO-BP analysis, enrichment was observed in the triglyceride metabolic process, acylglycerol metabolic process, and neutral lipid metabolic process (**Figure 10D**). In GO-CC analysis, enrichment included chylomicron and high- and low-density lipoproteins, and in GO-MF analysis, glycosaminoglycan binding and lipoprotein particle receptor binding were highlighted (**Figures 10E, F**). We then visualized the primary enrichment terms for each gene set and used t-SNE plots to graphically represent the risk score distribution of these enrichment terms (**Figure 10G**). GSEA results showed that the up-regulated genes were mainly enriched in processes such as intestinal absorption, peptidyl methionine modification, intestinal lipid absorption, and protein lipid complex assembly, while down-regulated genes were enriched in processes like regulation of release of sequestered calcium on into cytosol, external encapsulating structure organization, B cell receptor signaling pathway, and collagen fibril organization (**Figure 10H**). Additionally, we constructed a prognostic model to explore the clinical significance of MCs with high *SRSF7* expression. Univariate Cox regression analysis identified 11 genes significantly associated with prognosis (**Figure 10I**), with AHR as a protective factor (HR < 1) and the others as risk factors. To address the issue of multicollinearity among these genes, we further screened them using LASSO regression analysis, ultimately identifying eight prognostic-related genes (**Figure 10J**). Cox regression analysis was then used to calculate the coefficient values of these genes (**Figures 10K, L**). Curve and scatter plots demonstrated the differences in risk scores and survival outcomes between the two groups, indicating that the high SMRS group was associated with poorer prognosis (**Figure 10M**). Moreover, a heatmap displayed the differential expression patterns of genes used in model construction (**Figure 10N**). Kaplan-Meier survival curves further confirmed the conclusion that the high SMRS group had a worse survival outcome (**Figure 10O**). ROC curves and AUC values for 1-year, 3-year, and 5-year outcomes indicated that the model had good predictive value (**Figure 10P**).

## 4 Discussion

In recent years, the rapid development and application of scRNA-seq in cancer research has revolutionized our understanding of the biological features and dynamics within cancer lesions, greatly facilitating the diagnosis, treatment, and prognosis prediction of a range of tumors (59–61). Overall, the present study focused on mast cells in esophageal cancer, and we validated the pro-carcinogenic role of this pathway by launching a comprehensive profiling of mast cell subtypes with an eye on the C1 *SRSF7+* MCs and obtaining its reciprocal receptor, *EGFR*, using cellular communication analysis, and subsequently verifying the pro-carcinogenic role of this pathway through cellular knockdown experiments. In this study, we comprehensively characterized the cellular heterogeneity of EC using scRNA-seq technology. We identified immune cells including T-NK cells, MCs, and myeloid cells and so on, as well as non-immune cells such as smooth muscle cells and neuronal cells. In addition, we carefully analyzed the sample origin of these cell types and the distribution characteristics during the phase. Among them, MCs caught our attention. Until more than a hundred years ago, MCs were regarded as effectors of allergy, and it is only in the last two decades that MCs have gained recognition for their involvement in other physiological and pathological processes. MCs maturation, phenotype and function as a direct result of the local microenvironment (62), and by releasing a range of bioactive mediators has a significant effect on their ability to specifically recognize and respond to a variety of strategies (63–65). Therefore, depicting and analyzing the TME is important for MCs. And in previous studies, MCs have been shown to correlate with pro-tumorigenic effects (66–68). Despite the accumulating evidence for MCs in tumors, their exact role in the TME remains incompletely understood (51). We therefore focused our attention on the study of MCs. By further dimensionality reduction clustering, we obtained eight MCs subtypes, i.e., C0 *EGR1*+ MCs, C1 *SRSF7*+ MCs, C3 *TXNIP*+ MCs, C4 *S100A8*+ MCs, C5 *HSPA6*+ MCs, C6 *IL32*+ MCs, and C7 *RPL35A*+ MCs.

By integrating the proportions of MCs subtypes in sample sources and cell phases, Ro/e preference analyses, cell stemness analyses, and slingshot proposed pseudotime analyses, we identified the target subtype in this study: the C1 *SRSF7*+ MCs. C1 *SRSF7*+ MCs were significantly more abundant in tumor tissues than in pericancer tissues in P1 and P3 samples, and this was confirmed by Ro/e preference analysis. In slingshot proposed pseudotime analysis, Lineage 2 was considered to be representative of the differentiation trajectory of MCs associated with tumors. And the endpoint of Lineage 2 was a subtype of C1 *SRSF7*+ MCs, this result may prove that MCs are affected by some cytokines or tumor cell-secreted proteins during their development in the TME, leading to the transformation of MCs into a tumor-associated or pro-tumor phenotype, which is in line with the previous study (69). Meanwhile, cell stemness analysis by AUC value and CytoTRACE showed that the C1 *SRSF7*+ MCs subtype had the strongest cell stemness among all subtypes, with high differentiation potential, which did not contradict slingshot’s results, and it is understandable that the transformation from normal phenotype to TAMCs phenotype would result in an increase in cell stemness. It can be seen that the C1 *SRSF7*+ MCs subtype is intricately linked to tumor progression.

To further investigate the tumor-promoting related roles of the C1 *SRSF7*+ MCs subtype, we performed enrichment analysis and obtained the upregulated genes *DDX5*, T*PSB2*, and *CPA3*, of which *DDX5* interacts with a variety of key pro-tumorigenic molecules and participates in tumorigenic and tumor progression signaling pathways, and when *DDX5* is expressed or its post-translational modifications are deregulated, the normal cellular signaling network collapses, leading to many pathological states, including tumorigenesis and tumor progression (52, 70). Moreover, the enriched pathways obtained by GO-BP and GSEA on the C1 *SRSF7*+ MCs subtype showed that the C1 *SRSF7*+ MCs subtype was extensively involved in protein folding and refolding, regulation of immune system processes, and response to external stimuli. All these pathways suggest that the C1 *SRSF7*+ MCs subtype has probably been transformed into TAMCs. Finally, combining the above up-regulated genes and enriched pathways, we suggest that the C1 *SRSF7*+ MCs subtype is affected by the endoplasmic reticulum stress state (71), which disrupts the original protein equilibrium (72) and produces aberrant protein folding (73, 74), and this stress state dynamically reprograms the function of MCs, transforming MCs into TAMCs, which exerts pro-tumorigenic effects (75) and confers cancer cells with enhanced tumorigenic, metastatic, and drug-resistant capabilities. In this regard, we can treat patients with esophageal cancer by targeting the abnormal protein folding to prevent MCs from entering the endoplasmic reticulum stress state in patients, thus preventing the conversion of MCs into TAMCs, and thus controlling the progression of the cancer.

In addition, gene regulatory network of C1 *SRSF7*+ MCs was revealed by scenic analysis, in which the most valuable key regulators were ATF4 and JUNB. ATF4 showed a dual role in iron death and cancer under endoplasmic reticulum stress (75), and under sustained stress conditions, ATF4 promotes apoptotic cell death induction. Characterizing the mechanisms that regulate ATF4-mediated transcription and its effects on cellular metabolism may identify novel targets for cancer therapy (56). As for JUNB, more and more studies have shown that it is involved in tumorigenesis by regulating cell proliferation, differentiation, senescence, and metastasis, and in particular, it affects the TME by transcriptionally promoting or repressing oncogenes in tumor cells or immune cells (76). Furthermore, previous mechanistic studies have shown that JUNB overexpression regulates the mitochondrial apoptosis pathway, mediating resistance to FasL and TRAIL-induced cell death, and thus tumor resistance to immunotherapy (77). This study of ours provides a theoretical basis for subsequent analysis of drug sensitivity and provides new insights into the development of innovative targeted therapeutics.

To explore the interactions involving the C1 *SRSF7*+ mast cell subtype and other cell types, we employed CellChat communication pattern analysis. This approach helped reveal coordinated responses and interactions between different cell types in the context of their communication pathways. Different cell types can simultaneously activate common cell type-independent signaling pathways or different cell type-specific signaling transduction transduction pathways (77). Through CellChat analysis, we established the intercellular communication network between most cells, including tumor cells, fibroblasts, T-NK cells, and various subtypes of MCs, etc., as a way to characterize the relationship between the subtype of C1 *SRSF7*+ MCs and other cell types, and at the same time, we identified the three modes of outgoing, incoming and their corresponding signaling pathway expression. The C1 *SRSF7*+ MCs subtype belongs to mode 1 in the outgoing pathway, and its communication molecules, i.e., ligands, include ANNEXIN, PARs, CSF, ICAM, etc.; and it belongs to mode 2 in the incoming pathway, and its communication molecules, i.e., receptors, include BAFF, CLEC, ALCAM, SELPG, etc. It is also worth noting that tumor cells, which can be learned after our careful observation, belong to mode 2 on the outgoing and mode 1 on the incoming, echoing the subtype of C1 *SRSF7*+ MCs, which drew our attention.

By targeting tumor cells and the C1 *SRSF7*+ MCs subtype for interactions analysis, we have identified the secretion of AREG ligands by a subtype of C1 *SRSF7*+ MCs in the EGF signaling pathway that act on the protein receptor *EGFR* on the membrane of the tumor cells. In previous studies, the *EGFR* family has been validated to play a key role in *EGFR* signaling through the activation of many important cellular processes, including cell division, growth, and differentiation. Playing a key role in mediating cell growth factor signaling (78), overexpression of *EGFR* signaling widely promotes tumor progression and leads to promotion of proliferation and inhibition of apoptosis (79). And cancer immunotherapies, particularly immune checkpoint blockade (ICB), have transformed oncology care over the past decade and significantly improved survival in a wide range of metastatic tumors. Based on significant treatment benefits, ICB therapy is approved by the FDA as monotherapy or in combination with other cancer therapies for cancers such as melanoma, breast cancer, renal cell carcinoma, head and neck squamous cell carcinoma, and lung cancer (80–84). However, the MCs-mediated pro-tumor axis AREG-EGFR in EC has not yet been mentioned. Therefore, our study provides new EC target therapeutic approaches and provides a scientific basis for the treatment and prognosis of EC. Meanwhile, to further investigate the role of *EGFR* in EC, we performed *in vitro* experiments using TE-10 and KYSE-30 cell lines. We observed that *EGFR* knockdown inhibited tumor cell activity, migration and proliferation, thereby suppressing tumor growth. However, previous studies have shown that epidermal growth factor receptor inhibitors (EGFRIs) produce a variety of dermatologic side effects in the majority of patients, and this targeted therapeutic regimen needs to be further refined (85).

Given their role in promoting tumor growth and immune evasion, mast cells are considered potential therapeutic targets. Contemporary therapeutic strategies may include the use of mast cell stabilizers, mast cell mediator inhibitors, or blocking mast cell recruitment to tumor tissues and organs.

Finally, we constructed a prognostic model to indicate that the higher the SMRS score, the worse the prognosis.

Our study will direct attention to MCs in the progression of esophageal cancer, trigger attention to them, and promote researchers’ understanding of the tumor microenvironment in esophageal cancer. At the same time, we discovered the communication pathway between the tumor and our target MCs subtype. Although EFGR antagonists are still proved to have certain side effects, we believe that the development of targeted therapy will be further advanced in the future. However, this study still has some limitations. The relatively small sample size chosen is one aspect, and secondly, we only performed transcriptomics studies and *in vitro* experiments. The analysis of mast cell in EC using SCENIC and AUCell in our article is well-founded though and provides a detailed understanding of the regulatory networks that drive mast cell behavior. However, to draw more reliable conclusions, these findings must be validated by further experiments and compared across different cancer types. Next, we will integrate *in vivo* and *in vitro* experiments to provide a more comprehensive validation.

In conclusion, the innovative features of our study lie in the use of high-resolution single-cell analysis technology, the construction of cell-cell interaction networks, the analysis of dynamic evolutionary trajectories, the identification of regulatory networks, and experimental verification, which provide new ideas for the targeted treatment of MCs in EC and new cell carriers for the development of *EGFR* targeted drugs. These will help to promote the in-depth development of the research on EC and provide new strategies for the disease.

## Data Availability

The original contributions presented in the study are included in the article/supplementary material. Further inquiries can be directed to the corresponding author.

## References

[1] Sung H Ferlay J Siegel RL Laversanne M Soerjomataram I Jemal A. Global cancer statistics 2020: GLOBOCAN estimates of incidence and mortality worldwide for 36 cancers in 185 countries. CA Cancer J Clin. (2021) 71:209–49. doi: 10.3322/caac.21660 33538338

[2] Chen W Zheng R Baade PD Zhang S Zeng H Bray F. Cancer statistics in China, 2015. CA Cancer J Clin. (2016) 66:115–32. doi: 10.3322/caac.21338 26808342

[3] Baba Y Nomoto D Okadome K Ishimoto T Iwatsuki M Miyamoto Y. Tumor immune microenvironment and immune checkpoint inhibitors in esophageal squamous cell carcinoma. Cancer Sci. (2020) 111:3132–41. doi: 10.1111/cas.14541 PMC7469863 32579769

[4] Siegel RL Wagle NS Cercek A Smith RA Jemal A. Colorectal cancer statistics, 2023. CA Cancer J Clin. (2023) 73:233–54. doi: 10.3322/caac.21772 36856579

[5] Smyth EC Lagergren J Fitzgerald RC Lordick F Shah MA Lagergren P. Oesophageal cancer. Nat Rev Dis Primers. (2017) 3:17048. doi: 10.1038/nrdp.2017.48 28748917 PMC6168059

[6] Morgan E Soerjomataram I Rumgay H Coleman HG Thrift AP Vignat J. The global landscape of esophageal squamous cell carcinoma and esophageal adenocarcinoma incidence and mortality in 2020 and projections to 2040: new estimates from GLOBOCAN 2020. Gastroenterology. (2022) 163:649–58. doi: 10.1053/j.gastro.2022.05.054 35671803

[7] Zhang Y. Epidemiology of esophageal cancer. World J Gastroenterol. (2013) 19:5598–606. doi: 10.3748/wjg.v19.i34.5598 PMC3769895 24039351

[8] Dong J Gao M Li L Pan X Chen SY Li J. Associations of dietary inflammatory potential with esophageal precancerous lesions and esophageal squamous-cell cancer: A cross-sectional study. Nutrients. (2023) 15(8):4078. doi: 10.3390/nu15184078 37764860 PMC10537352

[9] You Y Chen Y Wei M Tang M Lu Y Zhang Q. Mediation role of recreational physical activity in the relationship between the dietary intake of live microbes and the systemic immune-inflammation index: A real-world cross-sectional study. Nutrients. (2024) 16(6):777. doi: 10.3390/nu16060777 38542688 PMC10974920

[10] Dowling GP Daly GR Hegarty A Hembrecht S Bracken A Toomey S. Comment on: Predictive value of pretreatment circulating inflammatory response markers in the neoadjuvant treatment of breast cancer: meta-analysis. Br J Surg. (2024) 111(5):znae132. doi: 10.1093/bjs/znae187 38801441 PMC11129713

[11] Zhang R Lau L Wu P Yip HC Wong SH. Endoscopic diagnosis and treatment of esophageal squamous cell carcinoma. Methods Mol Biol. (2020) 2129:47–62. doi: 10.1007/978-1-0716-0377-2_5 32056169

[12] Tang H Wang H Fang Y Zhu JY Yin J Shen YX. Neoadjuvant chemoradiotherapy versus neoadjuvant chemotherapy followed by minimally invasive esophagectomy for locally advanced esophageal squamous cell carcinoma: a prospective multicenter randomized clinical trial. Ann Oncol. (2023) 34:163–72. doi: 10.1016/j.annonc.2022.10.508 36400384

[13] Davern M Donlon NE Power R Hayes C King R Dunne MR. The tumour immune microenvironment in oesophageal cancer. Br J Cancer. (2021) 125:479–94. doi: 10.1038/s41416-021-01331-y PMC8368180 33903730

[14] Nallasamy P Nimmakayala RK Parte S Are AC Batra SK Ponnusamy MP. Tumor microenvironment enriches the stemness features: the architectural event of therapy resistance and metastasis. Mol Cancer. (2022) 21:225. doi: 10.1186/s12943-022-01682-x 36550571 PMC9773588

[15] Dinh HQ Pan F Wang G Huang QF Olingy CE Wu ZY. Integrated single-cell transcriptome analysis reveals heterogeneity of esophageal squamous cell carcinoma microenvironment. Nat Commun. (2021) 12:7335. doi: 10.1038/s41467-021-27599-5 34921160 PMC8683407

[16] Zhang X Peng L Luo Y Zhang S Pu Y Chen Y. Dissecting esophageal squamous-cell carcinoma ecosystem by single-cell transcriptomic analysis. Nat Commun. (2021) 12:5291. doi: 10.1038/s41467-021-25539-x 34489433 PMC8421382

[17] Zheng Y Chen Z Han Y Han L Zou X Zhou B. Immune suppressive landscape in the human esophageal squamous cell carcinoma microenvironment. Nat Commun. (2020) 11:6268. doi: 10.1038/s41467-020-20019-0 33293583 PMC7722722

[18] Segura-Villalobos D Ramirez-Moreno IG Martinez-Aguilar M Ibarra-Sanchez A Munoz-Bello JO Anaya-Rubio I. Mast cell-tumor interactions: molecular mechanisms of recruitment, intratumoral communication and potential therapeutic targets for tumor growth. Cells. (2022) 11(3):349. doi: 10.3390/cells11030349 35159157 PMC8834237

[19] Guo X Shen W Sun M Lv J Liu R. Activated mast cells combined with NRF2 predict prognosis for esophageal cancer. J Oncol. (2023) 2023:4211885. doi: 10.1155/2023/4211885 36644231 PMC9833916

[20] Puram SV Tirosh I Parikh AS Patel AP Yizhak K Gillespie S. Single-cell transcriptomic analysis of primary and metastatic tumor ecosystems in head and neck cancer. Cell. (2017) 171:1611–24. doi: 10.1016/j.cell.2017.10.044 PMC5878932 29198524

[21] Wagner J Rapsomaniki MA Chevrier S Anzeneder T Langwieder C Dykgers A. A single-cell atlas of the tumor and immune ecosystem of human breast cancer. Cell. (2019) 177:1330–45. doi: 10.1016/j.cell.2019.03.005 PMC6526772 30982598

[22] van Galen P Hovestadt V Wadsworth IM Hughes TK Griffin GK Battaglia S. Single-cell RNA-seq reveals AML hierarchies relevant to disease progression and immunity. Cell. (2019) 176:1265–81. doi: 10.1016/j.cell.2019.01.031 PMC6515904 30827681

[23] Liu S Chen LX Ye LS Hu B. Challenges in early detection and endoscopic resection of esophageal cancer: There is a long way to go. World J Gastrointest Oncol. (2024) 16:3364–67. doi: 10.4251/wjgo.v16.i7.3364 PMC11271785 39072158

[24] Zhang K Ye B Wu L Ni S Li Y Wang Q. Machine learning−based prediction of survival prognosis in esophageal squamous cell carcinoma. Sci Rep. (2023) 13:13532. doi: 10.1038/s41598-023-40780-8 37598277 PMC10439907

[25] Butler A Hoffman P Smibert P Papalexi E Satija R. Integrating single-cell transcriptomic data across different conditions, technologies, and species. Nat Biotechnol. (2018) 36:411–20. doi: 10.1038/nbt.4096 PMC6700744 29608179

[26] Xing J Cai H Lin Z Zhao L Xu H Song Y. Examining the function of macrophage oxidative stress response and immune system in glioblastoma multiforme through analysis of single-cell transcriptomics. Front Immunol. (2023) 14:1288137. doi: 10.3389/fimmu.2023.1288137 38274828 PMC10808540

[27] Jiang H Yu D Yang P Guo R Kong M Gao Y. Revealing the transcriptional heterogeneity of organ-specific metastasis in human gastric cancer using single-cell RNA Sequencing. Clin Transl Med. (2022) 12:e730. doi: 10.1002/ctm2.730 35184420 PMC8858624

[28] Wu F Fan J He Y Xiong A Yu J Li Y. Single-cell profiling of tumor heterogeneity and the microenvironment in advanced non-small cell lung cancer. Nat Commun. (2021) 12:2540. doi: 10.1038/s41467-021-22801-0 33953163 PMC8100173

[29] Ding Y Zhao Z Cai H Zhou Y Chen H Bai Y. Single-cell sequencing analysis related to sphingolipid metabolism guides immunotherapy and prognosis of skin cutaneous melanoma. Front Immunol. (2023) 14:1304466. doi: 10.3389/fimmu.2023.1304466 38077400 PMC10701528

[30] Ge Q Zhao Z Li X Yang F Zhang M Hao Z. Deciphering the suppressive immune microenvironment of prostate cancer based on CD4+ regulatory T cells: Implications for prognosis and therapy prediction. Clin Transl Med. (2024) 14:e1552. doi: 10.1002/ctm2.1552 38239097 PMC10797244

[31] Zhou Y Yang D Yang Q Lv X Huang W Zhou Z. Single-cell RNA landscape of intratumoral heterogeneity and immunosuppressive microenvironment in advanced osteosarcoma. Nat Commun. (2020) 11:6322. doi: 10.1038/s41467-020-20059-6 33303760 PMC7730477

[32] Korsunsky I Millard N Fan J Slowikowski K Zhang F Wei K. Fast, sensitive and accurate integration of single-cell data with Harmony. Nat Methods. (2019) 16:1289–96. doi: 10.1038/s41592-019-0619-0 PMC6884693 31740819

[33] Liu P Xing N Xiahou Z Yan J Lin Z Zhang J. Unraveling the intricacies of glioblastoma progression and recurrence: insights into the role of NFYB and oxidative phosphorylation at the single-cell level. Front Immunol. (2024) 15:1368685. doi: 10.3389/fimmu.2024.1368685 38510250 PMC10950940

[34] Zhou W Lin Z Tan W. Deciphering the molecular landscape: integrating single-cell transcriptomics to unravel myofibroblast dynamics and therapeutic targets in clear cell renal cell carcinomas. Front Immunol. (2024) 15:1374931. doi: 10.3389/fimmu.2024.1374931 38562930 PMC10982338

[35] Shao W Lin Z Xiahou Z Zhao F Xu J Liu X. Single-cell RNA sequencing reveals that MYBL2 in Malignant epithelial cells is involved in the development and progression of ovarian cancer. Front Immunol. (2024) 15:1438198. doi: 10.3389/fimmu.2024.1438198 39136009 PMC11317301

[36] Lin Z Sui X Jiao W Chen C Zhang X Zhao J. Mechanism investigation and experiment validation of capsaicin on uterine corpus endometrial carcinoma. Front Pharmacol. (2022) 13:953874. doi: 10.3389/fphar.2022.953874 36210802 PMC9532580

[37] Lin Z Li X Shi H Cao R Zhu L Dang C. Decoding the tumor microenvironment and molecular mechanism: unraveling cervical cancer subpopulations and prognostic signatures through scRNA-Seq and bulk RNA-seq analyses. Front Immunol. (2024) 15:1351287. doi: 10.3389/fimmu.2024.1351287 38482016 PMC10933018

[38] Zheng L Qin S Si W Wang A Xing B Gao R. Pan-cancer single-cell landscape of tumor-infiltrating T cells. Science. (2021) 374:abe6474. doi: 10.1126/science.abe6474 34914499

[39] Van den Berge K Roux DBH Street K Saelens W Cannoodt R Saeys Y. Trajectory-based differential expression analysis for single-cell sequencing data. Nat Commun. (2020) 11:1201. doi: 10.1038/s41467-020-14766-3 32139671 PMC7058077

[40] Aibar S Gonzalez-Blas CB Moerman T Huynh-Thu VA Imrichova H Hulselmans G. SCENIC: single-cell regulatory network inference and clustering. Nat Methods. (2017) 14:1083–86. doi: 10.1038/nmeth.4463 PMC5937676 28991892

[41] Gulati GS Sikandar SS Wesche DJ Manjunath A Bharadwaj A Berger MJ. Single-cell transcriptional diversity is a hallmark of developmental potential. Science. (2020) 367:405–11. doi: 10.1126/science.aax0249 PMC7694873 31974247

[42] Alexa A Rahnenfuhrer J Lengauer T. Improved scoring of functional groups from gene expression data by decorrelating GO graph structure. Bioinformatics. (2006) 22:1600–07. doi: 10.1093/bioinformatics/btl140 16606683

[43] Lin Z Fan W Yu X Liu J Liu P. Research into the mechanism of intervention of SanQi in endometriosis based on network pharmacology and molecular docking technology. Med (Baltimore). (2022) 101:e30021. doi: 10.1097/MD.0000000000030021 PMC9478308 36123943

[44] Zhao J Jiao W Sui X Zou J Wang J Lin Z. Construction of a prognostic model of luteolin for endometrial carcinoma. Am J Transl Res. (2023) 15:2122–39. PMC10086912 37056832

[45] Jin S Guerrero-Juarez CF Zhang L Chang I Ramos R Kuan CH. Inference and analysis of cell-cell communication using CellChat. Nat Commun. (2021) 12:1088. doi: 10.1038/s41467-021-21246-9 33597522 PMC7889871

[46] Lin Z Zou J Sui X Yao S Lin L Wang J. Necroptosis-related lncRNA signature predicts prognosis and immune response for cervical squamous cell carcinoma and endocervical adenocarcinomas. Sci Rep. (2022) 12:16285. doi: 10.1038/s41598-022-20858-5 36175606 PMC9523019

[47] Zou J Lin Z Jiao W Chen J Lin L Zhang F. A multi-omics-based investigation of the prognostic and immunological impact of necroptosis-related mRNA in patients with cervical squamous carcinoma and adenocarcinoma. Sci Rep. (2022) 12:16773. doi: 10.1038/s41598-022-20566-0 36202899 PMC9537508

[48] Zhao J Zou J Jiao W Lin L Wang J Lin Z. Construction of N-7 methylguanine-related mRNA prognostic model in uterine corpus endometrial carcinoma based on multi-omics data and immune-related analysis. Sci Rep. (2022) 12:18813. doi: 10.1038/s41598-022-22879-6 36335189 PMC9637130

[49] Lin Z Sui X Jiao W Wang Y Zhao J. Exploring the mechanism and experimental verification of puerarin in the treatment of endometrial carcinoma based on network pharmacology and bioinformatics analysis. BMC Complement Med Ther. (2022) 22:150. doi: 10.1186/s12906-022-03623-z 35672846 PMC9175360

[50] Lin Z Fan W Sui X Wang J Zhao J. Necroptosis-related lncRNA signatures for prognostic prediction in uterine corpora endometrial cancer. Reprod Sci. (2023) 30:576–89. doi: 10.1007/s43032-022-01023-9 PMC9988759 35854199

[51] Liu J Zhang Y Zhao J Yang Z Li D Katirai F. Mast cell: insight into remodeling a tumor microenvironment. Cancer Metastasis Rev. (2011) 30:177–84. doi: 10.1007/s10555-011-9276-1 21267769

[52] Xu K Sun S Yan M Cui J Yang Y Li W. DDX5 and DDX17-multifaceted proteins in the regulation of tumorigenesis and tumor progression. Front Oncol. (2022) 12:943032. doi: 10.3389/fonc.2022.943032 35992805 PMC9382309

[53] Cubillos-Ruiz JR Bettigole SE Glimcher LH. Tumorigenic and immunosuppressive effects of endoplasmic reticulum stress in cancer. Cell. (2017) 168:692–706. doi: 10.1016/j.cell.2016.12.004 28187289 PMC5333759

[54] Mukherjee D Bercz LS Torok MA Mace TA. Regulation of cellular immunity by activating transcription factor 4. Immunol Lett. (2020) 228:24–34. doi: 10.1016/j.imlet.2020.09.006 33002512 PMC7688477

[55] Wang HC Zhou Y Huang SK. SHP-2 phosphatase controls aryl hydrocarbon receptor-mediated ER stress response in mast cells. Arch Toxicol. (2017) 91:1739–48. doi: 10.1007/s00204-016-1861-1 27709270

[56] Wortel I van der Meer LT Kilberg MS van Leeuwen FN. Surviving stress: modulation of ATF4-mediated stress responses in normal and Malignant cells. Trends Endocrinol Metab. (2017) 28:794–806. doi: 10.1016/j.tem.2017.07.003 28797581 PMC5951684

[57] Han S Zhu L Zhu Y Meng Y Li J Song P. Targeting ATF4-dependent pro-survival autophagy to synergize glutaminolysis inhibition. Theranostics. (2021) 11:8464–79. doi: 10.7150/thno.60028 PMC8343999 34373753

[58] Beck A Shatz-Azoulay H Vinik Y Isaac R Boura-Halfon S Zick Y. Nedd4 family interacting protein 1 (Ndfip1) promotes death of pancreatic beta cells. Biochem Biophys Res Commun. (2015) 465:851–56. doi: 10.1016/j.bbrc.2015.08.099 26319551

[59] Gu X Cai L Luo Z Shi L Peng Z Sun Y. Identification and validation of a muscle failure index to predict prognosis and immunotherapy in lung adenocarcinoma through integrated analysis of bulk and single-cell RNA sequencing data. Front Immunol. (2022) 13:1057088. doi: 10.3389/fimmu.2022.1057088 36733390 PMC9888242

[60] Luo Z He Z Qin H Chen Y Qi B Lin J. Exercise-induced IL-15 acted as a positive prognostic implication and tumor-suppressed role in pan-cancer. Front Pharmacol. (2022) 13:1053137. doi: 10.3389/fphar.2022.1053137 36467072 PMC9712805

[61] Lei Y Tang R Xu J Wang W Zhang B Liu J. Applications of single-cell sequencing in cancer research: progress and perspectives. J Hematol Oncol. (2021) 14:91. doi: 10.1186/s13045-021-01105-2 34108022 PMC8190846

[62] Da SE Jamur MC Oliver C. Mast cell function: a new vision of an old cell. J Histochem Cytochem. (2014) 62:698–738. doi: 10.1369/0022155414545334 25062998 PMC4230976

[63] Dery RE Lin TJ Befus AD Milne CD Moqbel R Menard G. Redundancy or cell-type-specific regulation? Tumour necrosis factor in alveolar macrophages and mast cells. Immunology. (2000) 99:427–34. doi: 10.1046/j.1365-2567.2000.00982.x PMC2327162 10712673

[64] Blair RJ Meng H Marchese MJ Ren S Schwartz LB Tonnesen MG. Human mast cells stimulate vascular tube formation. Tryptase is a novel, potent angiogenic factor. J Clin Invest. (1997) 99:2691–700. doi: 10.1172/JCI119458 PMC508115 9169499

[65] Marech I Ammendola M Sacco R Capriuolo GS Patruno R Rubini R. Serum tryptase, mast cells positive to tryptase and microvascular density evaluation in early breast cancer patients: possible translational significance. BMC Cancer. (2014) 14:534. doi: 10.1186/1471-2407-14-534 25056597 PMC4117953

[66] Aller MA Arias A Arias JI Arias J. Carcinogenesis: the cancer cell-mast cell connection. Inflammation Res. (2019) 68:103–16. doi: 10.1007/s00011-018-1201-4 30460391

[67] Gorzalczany Y Sagi-Eisenberg R. Role of mast cell-derived adenosine in cancer. Int J Mol Sci. (2019) (10):2603. doi: 10.3390/ijms20102603 31137883 PMC6566897

[68] Alda S Ceausu RA Gaje PN Raica M Cosoroaba RM. Mast cell: A mysterious character in skin cancer. In Vivo. (2024) 38:58–68. doi: 10.21873/invivo.13410 38148067 PMC10756458

[69] Lichterman JN Reddy SM. Mast cells: A new frontier for cancer immunotherapy. Cells. (2021) 10(6):1270. doi: 10.3390/cells10061270 34063789 PMC8223777

[70] Nyamao RM Wu J Yu L Xiao X Zhang FM. Roles of DDX5 in the tumorigenesis, proliferation, differentiation, metastasis and pathway regulation of human Malignancies. Biochim Biophys Acta Rev Cancer. (2019) 1871:85–98. doi: 10.1016/j.bbcan.2018.11.003 30419318

[71] Oakes SA Papa FR. The role of endoplasmic reticulum stress in human pathology. Annu Rev Pathol. (2015) 10:173–94. doi: 10.1146/annurev-pathol-012513-104649 PMC5568783 25387057

[72] Mercier R LaPointe P. The role of cellular proteostasis in antitumor immunity. J Biol Chem. (2022) 298:101930. doi: 10.1016/j.jbc.2022.101930 35421375 PMC9108985

[73] Van Drie JH. Protein folding, protein homeostasis, and cancer. Chin J Cancer. (2011) 30:124–37. doi: 10.5732/cjc.010.10162 PMC4013342 21272445

[74] Scott MD Frydman J. Aberrant protein folding as the molecular basis of cancer. Methods Mol Biol. (2003) 232:67–76. doi: 10.1385/1-59259-394-1:67 12840540

[75] Visciano C Prevete N Liotti F Marone G. Tumor-associated mast cells in thyroid cancer. Int J Endocrinol. (2015) 2015:705169. doi: 10.1155/2015/705169 26379707 PMC4563106

[76] Ren FJ Cai XY Yao Y Fang GY. JunB: a paradigm for Jun family in immune response and cancer. Front Cell Infect Microbiol. (2023) 13:1222265. doi: 10.3389/fcimb.2023.1222265 37731821 PMC10507257

[77] Joung J Kirchgatterer PC Singh A Cho JH Nety SP Larson RC. CRISPR activation screen identifies BCL-2 proteins and B3GNT2 as drivers of cancer resistance to T cell-mediated cytotoxicity. Nat Commun. (2022) 13:1606. doi: 10.1038/s41467-022-29205-8 35338135 PMC8956604

[78] Kyriakopoulou K Kefali E Piperigkou Z Bassiony H Karamanos NK. Advances in targeting epidermal growth factor receptor signaling pathway in mammary cancer. Cell Signal. (2018) 51:99–109. doi: 10.1016/j.cellsig.2018.07.010 30071291

[79] Ayati A Moghimi S Salarinejad S Safavi M Pouramiri B Foroumadi A. A review on progression of epidermal growth factor receptor (EGFR) inhibitors as an efficient approach in cancer targeted therapy. Bioorg Chem. (2020) 99:103811. doi: 10.1016/j.bioorg.2020.103811 32278207

[80] Larkin J Chiarion-Sileni V Gonzalez R Grob JJ Cowey CL Lao CD. Combined nivolumab and ipilimumab or monotherapy in untreated melanoma. N Engl J Med. (2015) 373:23–34. doi: 10.1056/NEJMoa1504030 26027431 PMC5698905

[81] Schmid P Adams S Rugo HS Schneeweiss A Barrios CH Iwata H. Atezolizumab and nab-paclitaxel in advanced triple-negative breast cancer. N Engl J Med. (2018) 379:2108–21. doi: 10.1056/NEJMoa1809615 30345906

[82] Motzer RJ Rini BI McDermott DF Aren FO Hammers HJ Carducci MA. Nivolumab plus ipilimumab versus sunitinib in first-line treatment for advanced renal cell carcinoma: extended follow-up of efficacy and safety results from a randomised, controlled, phase 3 trial. Lancet Oncol. (2019) 20:1370–85. doi: 10.1016/S1470-2045(19)30413-9 PMC7497870 31427204

[83] Burtness B Harrington KJ Greil R Soulieres D Tahara M de Castro GJ. Pembrolizumab alone or with chemotherapy versus cetuximab with chemotherapy for recurrent or metastatic squamous cell carcinoma of the head and neck (KEYNOTE-048): a randomised, open-label, phase 3 study. Lancet. (2019) 394:1915–28. doi: 10.1016/S0140-6736(19)32591-7 31679945

[84] Gandhi L Rodriguez-Abreu D Gadgeel S Esteban E Felip E De Angelis F. Pembrolizumab plus chemotherapy in metastatic non-small-cell lung cancer. N Engl J Med. (2018) 378:2078–92. doi: 10.1056/NEJMoa1801005 29658856

[85] Lacouture ME Anadkat MJ Bensadoun RJ Bryce J Chan A Epstein JB. Clinical practice guidelines for the prevention and treatment of EGFR inhibitor-associated dermatologic toxicities. Support Care Cancer. (2011) 19:1079–95. doi: 10.1007/s00520-011-1197-6 PMC3128700 21630130

[86] Tang H Kang R Liu J Tang D. ATF4 in cellular stress, ferroptosis, and cancer. Arch Toxicol. (2024) 98:1025–41. doi: 10.1007/s00204-024-03681-x 38383612

