# Corrigendum: Unraveling the ecological landscape of mast cells in esophageal cancer through single-cell RNA sequencing

**DOI:** 10.3389/fimmu.2024.1522632

**Published:** 2024-11-15

**Authors:** Shengyi Zhang, Xinyi Zhang, Zhikai Xiahou, Shunqing Zuo, Jialong Xue, Yi Zhang

**Affiliations:** ^1^ Department of Thoracic Surgery, Songjiang Hospital Affiliated to Shanghai Jiao Tong University School of Medicine, Shanghai, China; ^2^ Clinical Medical College, Southwest Medical University, Luzhou, China; ^3^ China Institute of Sport and Health Science, Beijing Sport University, Beijing, China

**Keywords:** single-cell RNA sequencing, mast cells, EGFR signaling pathway, prognostic model, esophageal cancer

In the published article, there was an error in affiliation 1. Instead of “Songjiang Hospital Affiliated to Shanghai Jiao Tong University School of Medicine, Shanghai, 201600, China.”, it should be “Department of Thoracic Surgery, Songjiang Hospital Affiliated to Shanghai Jiao Tong University School of Medicine, Shanghai, 201600, China.”.

The authors apologize for this error and state that this does not change the scientific conclusions of the article in any way. The original article has been updated.

